# Three New 2-(2-Phenylethyl)chromone Derivatives of Agarwood Originated from *Gyrinops salicifolia*

**DOI:** 10.3390/molecules24030576

**Published:** 2019-02-06

**Authors:** Wen-Hua Dong, Hao Wang, Feng-Juan Guo, Wen-Li Mei, Hui-Qin Chen, Fan-Dong Kong, Wei Li, Kai-Bing Zhou, Hao-Fu Dai

**Affiliations:** 1 Institute of Tropical Agriculture and Forestry, Hainan University, Haikou 570228, China; dongwenhua@itbb.org.cn; 2 Key Laboratory of Biology and Genetic Resources of Tropical Crops, Ministry of Agriculture, Institute of Tropical Bioscience and Biotechnology, Chinese Academy of Tropical Agricultural Sciences, Haikou 571101, China; wanghao@itbb.org.cn (H.W.); guofengjuan0210@sina.com (F.-J.G.); meiwenli@itbb.org.cn (W.-L.M.); chenhuiqin@itbb.org.cn (H.-Q.C.); kongfandong@itbb.org.cn (F.-D.K.); liwei@itbb.org.cn (W.L.)

**Keywords:** agarwood, *Gyrinops salicifolia*, 2-(2-phenylethyl)chromone, acetylcholinesterase inhibitory, cytotoxicity

## Abstract

Two new 2-(2-phenylethyl)chromone derivatives (**1**–**2**), comprising 5,6,7,8-tetrahydro-2-(2-phenylethyl)chromone and benzylacetone moieties, together with one new 2-(2-phenylethenyl)chromone (**3**) were isolated from the ethyl acetate extraction of agarwood originated from *Gyrinops salicifolia* Ridl. All structures were unambiguously elucidated on the basis of 1D and 2D NMR spectra as well as by HRESIMS data. All isolated compounds were tested for acetylcholinesterase (AChE) inhibitory activity and cytotoxic activity against human myeloid leukemia cell line (K562). However, none of the compounds displayed AChE inhibitory activity at a concentration of 50 µg mL^−1^ or cytotoxic activity against K562 cell line.

## 1. Introduction

Agarwood, known as “Chenxiang” in Chinese, is the resinous heartwood of *Aquilaria* or *Gyrinops* genus (Thymelaeaceae) formed after various forms of natural or artificial injury [1]. As a rare traditional herbal medicine and natural spice, it possesses a panoply of effects such as aphrodisiac, sedative, cardiatonic and carminative activity, and it is used to relieve gastric problems, coughs, rheumatism and high fever [2]. Until now, the chemical constituents of agarwood harvested from *A. malassensis*, *A. sinensis*, *A. crassna*, *A. agallocha*, and *G. salicifolia* have been well or partly investigated. 2-(2-Phenylethyl)chromone derivatives and sesquiterpenes were reported as the main chemical constituents of agarwood and exhibited various of biological activities, including cytotoxicity, antibacterial, acetylcholinesterase (AChE) inhibitory, α-glucosidase inhibitory, antineuroinflammatory, neuroprotective and antidepressant activities [3,4,5,6,7,8].

**Figure 1:**
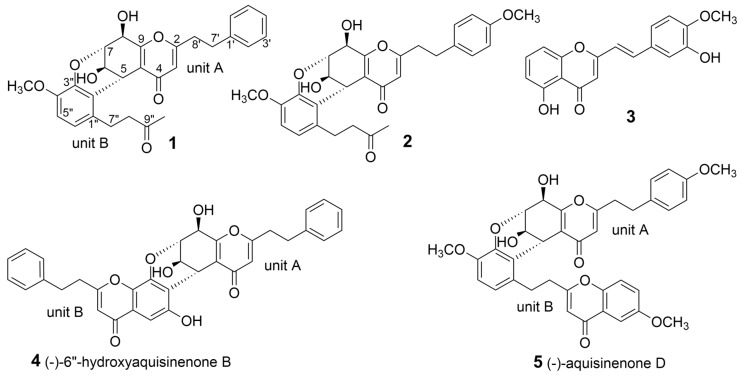
Chemical structures of 2-(2-phenylethyl)chromone derivatives **1**–**5**.

*Gyrinops salicifolia* Ridl. is one of agarwood-producing endemic species in Papua New Guinea. In our previous studies on new bioactive chemical constituents from *G. salicifolia*, several 2-(2-phenylethyl)chromones and sesquiterpenes were identified and showed cytotoxicity and AChE inhibitory activities [9,10]. In order to further explore the feature and active constituents of agarwood originating from *G. salicifolia*, contributing to the deeper understanding of the similarities and differences among agarwood, the investigation of ethyl acetate extraction of agarwood originated from *G. salicifolia* was continued and led to the identification of two new 2-(2-phenylethyl)chromone derivatives (**1**–**2**), comprising 2-(2-phenylethyl)chromone and benzylacetone moieties, and one new 2-(2-phenylethenyl)chromone (**3**) (Figure 1). Herein, this paper describes the isolation and elucidation of new compounds.

## 2. Results and Discussion

Chromatographic separation of ethyl acetate extraction of agarwood originated from *G. salicifolia* led to the isolation of three 2-(2-phenylethyl)chromone derivatives (**1**–**3**). Their structures were elucidated by HRESIMS and NMR spectroscopic analyses, the data as shown in Table 1 and Table 2. HRESIMS and NMR spectra for compounds **1**–**3** are shown in the Supplementary Materials.

Compound **1** was obtained as a yellow powder. Its molecular formula was deduced to be C_28_H_28_O_7_ with 15 degrees of unsaturation on the basis of the HRESIMS data. The ^1^H-NMR displayed a monosubstituted benzene ring at *δ*_H_ 7.10–7.25 (H-2′–6′), two doublet aromatic protons (*δ*_H_ 6.56, H-6″; 6.70, H-5″), the characteristic olefinic proton singlet of 2-(2-phenylethyl)chromone at *δ*_H_ 6.07 (H-3), four methines (*δ*_H_ 4.61, H-7; 4.33, H-8; 4.31, H-5; and 4.23, H-6), one methoxy group singlet at *δ*_H_ 3.64 (OCH_3_-4″), one methyl group singlet at *δ*_H_ 2.06 (H_3_-10″), and four methylene groups ranging from *δ*_H_ 2.40 to 3.50. The DEPTQ spectrum showed the presence of two carbonyls at *δ*_C_ 208.7 (C-9″), and 178.0 (C-4), two benzene rings, and four olefinic carbons, accounting for 12 degrees of unsaturation. Apart from these signals, four methines at *δ*_C_ 74.7 (C-7), 68.8 (C-8), 63.6 (C-6), and 31.7 (C-5), four methylene groups at *δ*_C_ 45.4 (C-8″), 34.3 (C-8′), 32.2 (C-7′), and 25.6 (C-7″), one methoxy group at *δ*_C_ 55.6 (OCH_3_-4″) and one methyl group at *δ*_C_ 30.0 (CH_3_-10″) were observed in DEPTQ spectrum of **1**. Detailed analysis of its 1D and 2D NMR data revealed that compound **1** was a dimer comprising a 5,6,7,8-tetrahydro-2-(2-phenylethyl)chromone unit and a benzylacetone unit. The 5,6,7,8-tetrahydro-2-(2-phenylethyl)chromone unit was identical to that of monomeric A of (−)-6″-hydroxyaquisinenone B by comparison of NMR data and key HMBC correlations from H-2′ and 6′ (*δ*_H_ 7.18) to C-7′, from H-3 to C-8′ and C-5, from H-5 to C-4, C-6 and C-7, from H-6 to C-10 (*δ*_C_ 121.7), from H-7 to C-9 (*δ*_C_ 162.5), from H-8 to C-9, C-10, C-6, and C-7, and ^1^H-^1^H COSY of H-6/H-7, H-2′/H-3′/H-4′/H-5′/H-6′, H-7′/H-8′ [11]. The structure of the benzylacetone unit was elucidated by ^1^H-^1^H COSY of H-5″/H-6″, H_2_-7″ (*δ*_H_ 2.76 and 3.48)/H_2_-8″ (*δ*_H_ 2.48 and 2.61), and HMBC correlations from H_3_-10″ to C-8″ and C-9″, from H_2_-7″ to C-8″, C-9″, C-1″ (*δ*_C_ 131.6), C-2″ (*δ*_C_ 123.0) and C-6″ (*δ*_C_ 120.9), and from H-6″ to C-2″, C-4″ (*δ*_C_ 146.1), and C-7″. The position of the methoxy group at C-4″ was elucidated by HMBC correlations from 4″-OCH_3_ to C-4″, and by NOE correlation between 4″-OCH_3_ and H-5″. The linkage between two units by C-5/C-2″ and C-7/O/C-3″ (*δ*_C_ 141.3) was determined by HMBC correlations from H-7 to C-3″, and from H-5 to C-1″, C-2″ and C-3″ as shown. Due to the formation of a 3,4-dihydro-2*H*-pyran ring between two units, H-5 and H-7, as equatorial hydrogens, were oriented towards the same face of the cyclohexene ring in a half-chair conformation, which was confirmed by “W” coupling between H-5 and H-7 (^4^*J*_5,7_ = 2.1 Hz). The *trans*-type relationships of H-7/H-8 was deduced by their small coupling constant (~90° dihedral angle of H_7_–C_7_–C_8_–H_8_). The remaining relative configuration of H-6 was elucidated as opposite to H-5 by its close ^3^*J*_5,6_ (2.1 Hz) to that of (−)-6″-hydroxyaquisinenone B (^3^*J*_5,6_ = 2.5 Hz) and ^3^*J*_6,7_ (3.4 Hz) to that of (+)-6″-hydroxy-4′,4′′′-dimethoxyaquisinenone B (^3^*J*_6,7_ = 3.5 Hz) [11]. The relative configuration of **1** was identical to that of (−)-6″-hydroxyaquisinenone B and (+)-6″-hydroxy-4′,4′′′-dimethoxyaquisinenone B by further detailed comparison of their coupling constants of H-5–8. Thus, the structure of **1** was established as depicted and named gyrinone A.

**Figure 2:**
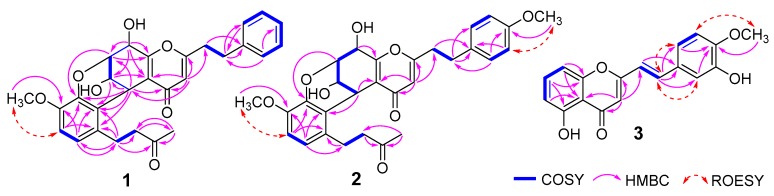
Key ^2^D-NMR correlations of 2-(2-phenylethyl)chromone derivatives **1**–**3** of agarwood.

Compound **2** was isolated as a yellow amorphous solid. It had the molecular formula C_29_H_30_O_8_ as established by HRESIMS, indicating the addition of a methoxy group compared to **1**. The ^1^H- and ^13^C-NMR spectra were similar to those of **1**, except for the presence of one more methoxy group. The ^1^H-NMR spectra of **1** revealed the presence of a *para*-disubstituted benzene ring (*δ*_H_ 6.76, H-3′/5′; and 7.07, H-2′/6′), suggested a methoxy group attached to C-4′ (*δ*_C_ 159.7). The deduction was confirmed by HMBC correlation from 4′-OCH_3_ (*δ*_H_ 3.73) to C-4′, and by NOE correlation from 4′-OCH_3_ to H-3′ and H-5′ (Figure 2). The remaining substructures of **2** were identical to those of **1** based on detailed analysis of 1D- and 2D-NMR spectra. In the same way to **1**, the relative configuration of **2** was identical to that of (−)-aquisinenone D (**5**) and **1** by analysis of their configuration and for their close chemical shifts of unit A and coupling constants of H-6 and H-8 (Table 1 and Table 2) [11]. Therefore, the structure of **2** was elucidated as shown (Figure 1) and named gyrinone B.

Compound **3** was obtained as yellow powder, and its molecular formula was deduced to be C_18_H_14_O_5_ on the basis of HRESIMS. Its ^1^H-NMR spectroscopic data showed two *trans*-olefinic protons at *δ*_H_ 7.54 (H-7′) and *δ*_H_ 6.61 (H-8′), a 1,2,3-trisubstituted benzene ring (*δ*_H_ 6.79, H-6; 7.52, H-7; and 6.96, H-8), a set of ABX coupling aromatic system at *δ*_H_ 7.10 (H-6′), 6.89 (H-5′) and 7.20 (H-2′), and a methoxy group at *δ*_H_ 3.95 (4′-OCH_3_). The ^1^H and ^13^C-NMR spectroscopic data of **3** were similar to those of 5-hydroxy-2-[2-(4-methoxybenzene)ethenyl]chromone [9], except for containing an additional hydroxy group in **3**. The HMBC correlations from 4′-OCH_3_, H-2′, and H-6′ to C-4′ (*δ*_C_ 148.5), together with NOE of 4′-OCH_3_ and H-5′, indicated that the methoxy group was attached to C-4′. Thus, C-5 (*δ*_C_ 160.9) and C-3′ (*δ*_C_ 146.1) were substituted by hydroxy groups based on their much further downfield chemical shifts. Finally, compound **3** was established to be 5-hydroxy-2-[2-(3-hydroxy-4-methoxyphenyl)ethenyl]chromone by a comprehensive analysis of its ^2^D-NMR data (Figure 2).

Compounds **1**–**3** were tested for AChE inhibitory activity in vitro and cytotoxicity against K562 human myeloid leukemia cell line. Unfortunately, none of the compounds displayed AChE inhibitory activity or cytotoxicity against K562 cell line.

## 3. Materials and Methods

### 3.1. General Procedures

^1^D-and ^2^D-NMR experiments were recorded on Bruker AVANCE IIITM 600 MHz or Bruker AVIII 500 MHz spectrometers (Bruker, Bremen, Germany). Chemical shifts were referenced to the solvent residual peaks. The HRESIMS were acquired using an API Qstar Pulsar mass spectrometer (Bruker, Bremen, Germany). Optical rotations were recorded on an MCP 5100 polorimeter (Anton Paar, Graz, Austria). IR spectra were measured on a Nicolet 380 FT-IR spectrometer (Thermo, Pittsburgh, PA, America). HPLC analysis was performed on Agilent Technologies 1260 Infinity II (Agilent, Palo Alto, CA, USA) with a reversed-phased column (YMC-packed C18, 5 μm, 250 mm × 10 mm) using a Dionex P680 pump and detected with a Dionex UVD 170 U detector (*λ* = 254 nm). Silica gel (60–80, 200–300 mesh, Qingdao Marine Chemical Co. Ltd., Qingdao, China), ODS gel (20–45 μm, Fujian Silysia Chemical Co. Ltd., Fuzhou, China) and Sephadex LH-20 (40–70 μm, Merck, Darmstadt, Germany) were used for column chromatography. TLC was carried out on silica gel G precoated plates (Qingdao Haiyang Chemical Co. Ltd., Qingdao, China), and the peaks on TLC were detected by a UV lamp at 254 nm and then sprayed with 5% H_2_SO_4_ in EtOH. The methanol used for HPLC analysis was of chromatographic grade (Concord Technology Co. Ltd., Tianjin, China). Tacrine hydrochloride hydrate (99%) and paclitaxel (99%) were purchased from Sigma Chemical.

### 3.2. Plant Material

The agarwood sample was collected in Papua New Guinea, then traded in Macao, one of China′s special administrative regions, in Dec. 2014, and identified as agarwood originating from *Gyrinops salicifolia* Ridl. by Prof. Dr. Hao-Fu Dai and Dr. Jun Wang (Institute of Tropical Bioscience and Biotechnology, Chinese Academy of Tropical Agricultural Sciences & Hainan engineering research center of agarwood). A voucher specimen (CX 20141222) has been deposited at the Institute of Tropical Bioscience and Biotechnology, Chinese Academy of Tropical Agricultural Sciences.

### 3.3. Extraction and Isolation

The dried agarwood sample (491.1 g) was extracted with 95% EtOH (2 L) for three times at heating reflux and filtered. After removing EtOH under reduced pressure, the crude extract (177.4 g) was obtained and then suspended in H_2_O (2 L), subsequently extracted with EtOAc (2 L), followed by *n*-BuOH (2 L). The EtOAc extract (141.2 g) was subjected to vacuum liquid chromatography with silica gel (10 × 55 cm) using a step gradient of CHCl_3_-MeOH (*v*/*v*, 1:0, 50:1, 25:1, 15:1, 10:1, 5:1, 2:1, 1:1, 0:1, 6 L of each) to yield 10 fractions (Fr.1~10). Fr.6 (27.2 g) was applied to ODS column (3 × 40 cm) chromatography with MeOH-H_2_O (*v*/*v*, 3:7, 4:6, 5:5, 6:4, 7:3, 8:2, 9:1, 1:0, 4 L of each) divided to 14 fractions (Fr.6-1~14). Fr.6-3 (85.2 mg) was submitted to Sephadex LH-20 (column: 1.2 × 50 cm) in MeOH to get Fr.6-3-1 (36.0 mg), then purified through silica gel column (1.2 × 40 cm) chromatography with CHCl_3_-MeOH (*v*/*v* = 60:1) to obtain compound **3** (3.5 mg). Fr.6-7 (182.9 mg) was separated on Sephadex LH-20 (petroleum ether:CHCl_3_:MeOH = 1:1:1), and then chromatographed on silica gel with CHCl_3_-MeOH (*v*/*v*, 100:1, 50:1) to afford compounds **1** (1.1 mg) and **2** (1.1 mg).

*Gyrinone A* (**1**): yellow powder; ${\left[\alpha \right]}_{D}^{25}$ = −8.4 (*c* 0.05, MeOH); UV (MeOH) 298, 224 nm; IR (KBr) *ν*_max_ 3434, 2977, 2922, 1672, 1635, 1400, 1384, 1048 cm^−1^; ^1^H- and ^13^C-NMR data, see Table 1 and Table 2; HRESIMS *m*/*z* 499.1730 [M + Na]^+^ (calcd. C_28_H_28_NaO_7_ for 499.1727).

*Gyrinone B* (**2**): yellow amorphous solid; ${\left[\alpha \right]}_{D}^{25}$ = −11.2 (*c* 0.04, MeOH); UV (MeOH) 297, 222 nm; IR (KBr) *ν*_max_ 3434, 2977, 2921, 1673, 1403, 1387, 1140, 1029 cm^−1^; ^1^H- and ^13^C-NMR data, see Table 1 and Table 2; HRESIMS *m*/*z* 529.1839 [M + Na]^+^ (calcd. C_29_H_30_NaO_8_ for 529.1833).

*5-Hydroxy-2-[2-(3-hydroxy-4-methoxyphenyl)ethenyl]chromone* (**3**): yellow powder; UV (MeOH) 383, 225 nm; IR (KBr) *ν*_max_ 3440, 2968, 1649, 1602, 1512, 1475, 1411, 1260, 1155, 1131, 1029, 961 cm^−1^; ^1^H- and ^13^C-NMR data, see Table 1 and Table 2; HRESIMS *m*/*z* 311.0923 [M + H]^+^ (calcd. C_18_H_15_O_5_ for 311.0914).

### 3.4. Bioassays

#### 3.4.1. Bioassay for AChE Inhibitory Activity In Vitro

All compounds were tested for AChE inhibitory activity by Ellman’s colorimetric method in vitro at a concentration of 50 µg mL^−1^ as described previously [12]. Tacrine hydrochloride hydrate was used as positive control with an IC_50_ value of 64.8 nM, and DMSO was served as negative control.

#### 3.4.2. Bioassay for Cytotoxic Activity

The MTT assay was used to evaluate cytotoxicity of all compounds against human myeloid leukemia cell line (K562) as described previously [9]. K562 cell line was obtained from the Cell Bank of Type Culture Collection of the Chinese Academy of Sciences, Shanghai Institute of Cell Biology. Paclitaxel was performed as positive control with an IC_50_ value of 0.89 µM.

## 4. Conclusions

Three 2-(2-phenylethyl)chromone derivatives (**1**–**3**) were isolated from agarwood originating from *Gyrinops salicifolia* Ridl. Gyrinones A and B, comprising 5,6,7,8-tetrahydro-2-(2-phenylethyl)chromone and benzylacetone units, were elucidated as novel structures. However, bioassay tests of all compounds showed that there was no inhibition effect on AChE inhibitory activity or cytotoxicity against K562 cell line.

## Figures and Tables

**Table 1:** ^1^H-NMR data for compounds **1**–**3** and unit as of (−)-6″-hydroxyaquisinenone B (**4**) and (−)-aquisinenone D (**5**) (*δ* in ppm, *J* in Hz).

Position	1 ^a^	Unit A of 4 ^b^	2 ^c^	Unit A of 5 ^d^	3 ^e^
3	6.07, s	6.42, s	6.05, s	6.02, s	6.22, s
5	4.31, t (2.1)	4.29, t (2.5)	4.53, dd (3.0, 1.9)	4.49, br s	
6	4.23, dd (3.4, 2.1)	4.40, m	4.41, dd (4.9, 3.0)	4.32, dd (4.5, 3.0)	6.79, d (8.3)
7	4.61, m	4.87, br s	4.74, dt (4.9, 1.9)	4.73, m	7.52, t (8.3)
8	4.33, br s	4.55, d (7.0)	4.47, d (1.9)	4.46, d (2.0)	6.96, d (8.3)
2′	7.18, d (7.2)	7.27, m	7.07, d (8.6)	7.06, d (8.5)	7.20, d (2.1)
3′	7.22, t (7.2)	7.27, m	6.76, d (8.6)	6.75, d (8.5)	
4′	7.14, t (7.2)	7.19, m			
5′	7.22, t (7.2)	7.27, m	6.76, d (8.6)	6.75, d (8.5)	6.89, d (8.3)
6′	7.18, d (7.2)	7.27, m	7.07, d (8.6)	7.06, d (8.5)	7.10, dd (8.3, 2.1)
7′	2.81, 2.89, m	2.99, m	2.84, 2.93, m	2.90, m	7.54, d (15.9)
8′	2.81, m	2.92, m	2.93, m	2.82, m	6.61, d (15.9)
5′′	6.70, d (8.3)		6.76, d (8.3)		
6′′	6.56, d (8.3)		6.66, d (8.3)		
7′′	2.76, 3.48, m		2.93, 3.64, m		
8′′	2.48, 2.61, m		2.61, 2.71, m		
10′′	2.06, s		2.18, s		
4′-OCH_3_			3.73, s		3.95, s
4″-OCH_3_	3.64, s		3.77, s		
6-OH		5.87, d (3.0)			
8-OH		6.11, d (8.0)			

^a^ Recorded at 600 MHz in DMSO-*d*_6_, ^b^ Recorded at 500 MHz in DMSO-*d*_6_, ^c^ Recorded at 600 MHz in CD_3_OD, ^d^ Recorded at 500 MHz in CD_3_OD, ^e^ Recorded at 500 MHz in CDCl_3_.

**Table 2:** ^13^C-NMR data for compounds **1**–**3** and unit as of (−)-6″-hydroxyaquisinenone B (**4**) and (−)-aquisinenone D (**5**) (*δ* in ppm).

Position	1 ^a^	Unit A of 4 ^b^	2 ^c^	Unit A of 5 ^d^	3 ^e^
2	168.0, C	170.1, C	170.7, C	170.6, C	163.3, C
3	112.7, CH	111.8, CH	113.8, CH	113.7, CH	108.5, CH
4	178.0, C	180.0, C	181.0, C	180.9, C	183.6, C
5	31.7, CH	29.3, CH	33.4, CH	33.4, CH	160.9, C
6	63.6, CH	61.3, CH	65.6, CH	65.5, CH	111.3, CH
7	74.7, CH	77.2, CH	75.6, CH	75.8, CH	135.3, CH
8	68.8, CH	68.1, CH	70.4, CH	70.3, CH	106.9, CH
9	162.5, C	164.1, C	164.3, C	164.3, C	156.3, C
10	121.7, C	121.1, C	122.9, C	122.8, C	111.0, C
1′	140.3, C	140.0, C	133.0, C	133.1, C	128.6, C
2′	128.6, CH	128.3, CH	130.4, CH	130.4, CH	112.8, CH
3′	128.7, CH	128.4, CH	114.9, CH	114.9, CH	146.1, C
4′	126.5, CH	126.2, CH	159.7, C	159.7, C	148.5, C
5′	128.7, CH	128.4, CH	114.9, CH	114.9, CH	110.8, CH
6′	128.6, CH	128.3, CH	130.4, CH	130.4, CH	121.7, CH
7′	32.2, CH_2_	32.1, CH_2_	33.1, CH_2_	33.1, CH_2_	138.0, CH
8′	34.3, CH_2_	34.6, CH_2_	36.5, CH_2_	36.5, CH_2_	117.9, CH
1′′	131.6, C		133.2, C		
2′′	123.0, C		123.9, C		
3′′	141.3, C		142.8, C		
4′′	146.1, C		147.6, C		
5′′	111.1, CH		112.4, CH		
6′′	120.9, CH		122.3, CH		
7′′	25.6, CH_2_		26.9, CH_2_		
8′′	45.4, CH_2_		46.6, CH_2_		
9′′	208.7, C		211.6, C		
10′′	30.0, CH_3_		30.0, CH_3_		
4′-OCH_3_			55.6, CH_3_		56.1, CH_3_
4″-OCH_3_	55.6, CH_3_		56.5, CH_3_		

^a^ Recorded at 150 MHz in DMSO-*d*_6_, ^b^ Recorded at 125 MHz in DMSO-*d*_6_, ^c^ Recorded at 150 MHz in CD_3_OD, ^d^ Recorded at 125 MHz in CD_3_OD, ^e^ Recorded at 125 MHz in CDCl_3_.

## Supplementary Materials

HRESIMS and NMR spectra for compounds **1**–**3** are available online.

## References

[1] Borris R.P., Blaskó G., Cordell G.A. (1988). Ethnopharmacologic and phytochemical studies of the Thymelaeaceae. J. Ethnopharmacol..

[2] Naef R. (2011). The volatile and semi-volatile constituents of agarwood, the infected heartwood of *Aquilaria* species: A review. Flavour. Fragr. J..

[3] Mei W.L., Zeng Y.B., Guo Z.K., Zhao Y.X., Wang H., Zuo W.J., Dong W.H., Wang Q.H., Dai H.F. (2013). 2-(2-phenylethyl)chromone derivatives in Chinese Agarwood “Qi-Nan” from *Aquilaria sinensis*. Planta Med..

[4] Li W., Cai C.H., Dong W.H., Guo Z.K., Wang H., Mei W.L., Dai H.F. (2014). 2-(2-Phenylethyl)chromone derivatives from Chinese agarwood induced by artificial holing. Fitoterapia.

[5] Ibrahim S.R.M., Mohamed G.A. (2015). Natural occurring 2-(2-phenylethyl) chromones, structure elucidation and biological activities. Nat. Prod. Res..

[6] Chen H.Q., Wei J.H., Yang J.S., Zhang Z., Yang Y., Gao Z.H., Sui C., Gong B. (2012). Chemical constituents of agarwood originating from the endemic genus *Aquilaria plants*. Chem. Biodivers..

[7] Yang D.L., Wang H., Guo Z.K., Li W., Mei W.L., Dai H.F. (2014). Fragrant agarofuran and eremophilane sesquiterpenes in agarwood “Qi-Nan” from *Aquilaria sinensis*. Phytochem. Lett..

[8] Li W., Cai C.H., Guo Z.K., Wang H., Zuo W.J., Dong W.H., Mei W.L., Dai H.F. (2015). Five new eudesmane-type sesquiterpenoids from Chinese agarwood induced by artificial holing. Fitoterapia.

[9] Shao H., Mei W.L., Dong W.H., Gai C.J., Li W., Zhu G.P., Dai H.F. (2016). 2-(2-Phenylethyl) chromone Derivatives of Agarwood Originating from *Gyrinops salicifolia*. Molecules.

[10] Shao H., Mei W.L., Kong F.D., Dong W.H., Gai C.J., Li W., Zhu G.P., Dai H.F. (2016). Sesquiterpenes of agarwood from *Gyrinops salicifolia*. Fitoterapia.

[11] Huo H.X., Zhu Z.X., Song Y.L., Shi S.P., Sun H., Zhao Y.F., Zheng J., Ferreira D., Zjawiony J.K., Tu P.F., Li J. (2017). Anti-inflammatory dimeric 2-(2-phenylethyl) chromones from the resinous wood of *Aquilaria sinensis*. J. Nat. Prod..

[12] Liao G., Mei W.L., Kong F.D., Li W., Yuan J.Z., Dai H.F. (2017). 5,6,7,8-Tetrahydro-2-(2-phenylethyl) chromones from artificial agarwood of *Aquilaria sinensis* and their inhibitory activity against acetylcholinesterase. Phytochemistry.

