# Supplementary material for: Three New 2-(2-Phenylethyl)chromone Derivatives of Agarwood Originated from Gyrinops salicifolia
Source: Molecules. 2019 Feb 6;24(3):576. doi: 10.3390/molecules24030576 (PMC6384947; doi:10.3390/molecules24030576)
Supplement: Supplementary file 1 [file molecules-24-00576-s001.pdf]

Table of Contents

Figure S1. HRESIMS spectrum of **1**……………………………………………………………1
Figure S2. ^{1}H NMR (600 MHz, DMSO-d_6) spectrum of **1**………………………………2
Figure S3. DEPTQ (150 MHz, DMSO-d_6) spectrum of **1**…………………………………3
Figure S4. ^{1}H-^{1}H COSY (600 MHz, DMSO-d_6) spectrum of **1**…………………………4
Figure S5. HSQC (600 and 150 MHz, DMSO-d_6) spectrum of **1**……………………………5
Figure S6. HMBC (600 and 150 MHz, DMSO-d_6) spectrum of **1**……………………………6
Figure S7. ROESY (600 MHz, DMSO-d_6) spectrum of **1**……………………………………7
Figure S8. HRESIMS spectrum of **2**……………………………………………………………8
Figure S9. ^{1}H NMR (600 MHz, methanol-d_4) spectrum of **2**……………………………9
Figure S10. ^{13}C NMR and DEPT-135 (150 MHz, methanol-d_4) spectrum of **2**…………10
Figure S11. ^{1}H-^{1}H COSY (600 MHz, methanol-d_4) spectrum of **2**……………………11
Figure S12. HSQC (600 and 150 MHz, methanol-d_4) spectrum of **2**…………………………12
Figure S13. HMBC (600 and 150 MHz, methanol-d_4) spectrum of **2**…………………………13
Figure S14. ROESY (600 MHz, methanol-d_4) spectrum of **2**…………………………………14
Figure S15. HRESIMS spectrum of **3**…………………………………………………………15
Figure S16. ^{1}H NMR (500 MHz, $CDCl_3$) spectrum of **3**…………………………………16
Figure S17. ^{13}C NMR and DEPT-135 (125 MHz, $CDCl_3$) spectrum of **3**………………17
Figure S18. ^{1}H-^{1}H COSY (500 MHz, $CDCl_3$) spectrum of **3**……………………………18
Figure S19. HSQC (500 and 125 MHz, $CDCl_3$) spectrum of **3**………………………………19
Figure S20. HMBC (500 and 125 MHz, $CDCl_3$) spectrum of **3**………………………………20
Figure S21. ROESY (500 MHz, $CDCl_3$) spectrum of **3**………………………………………21

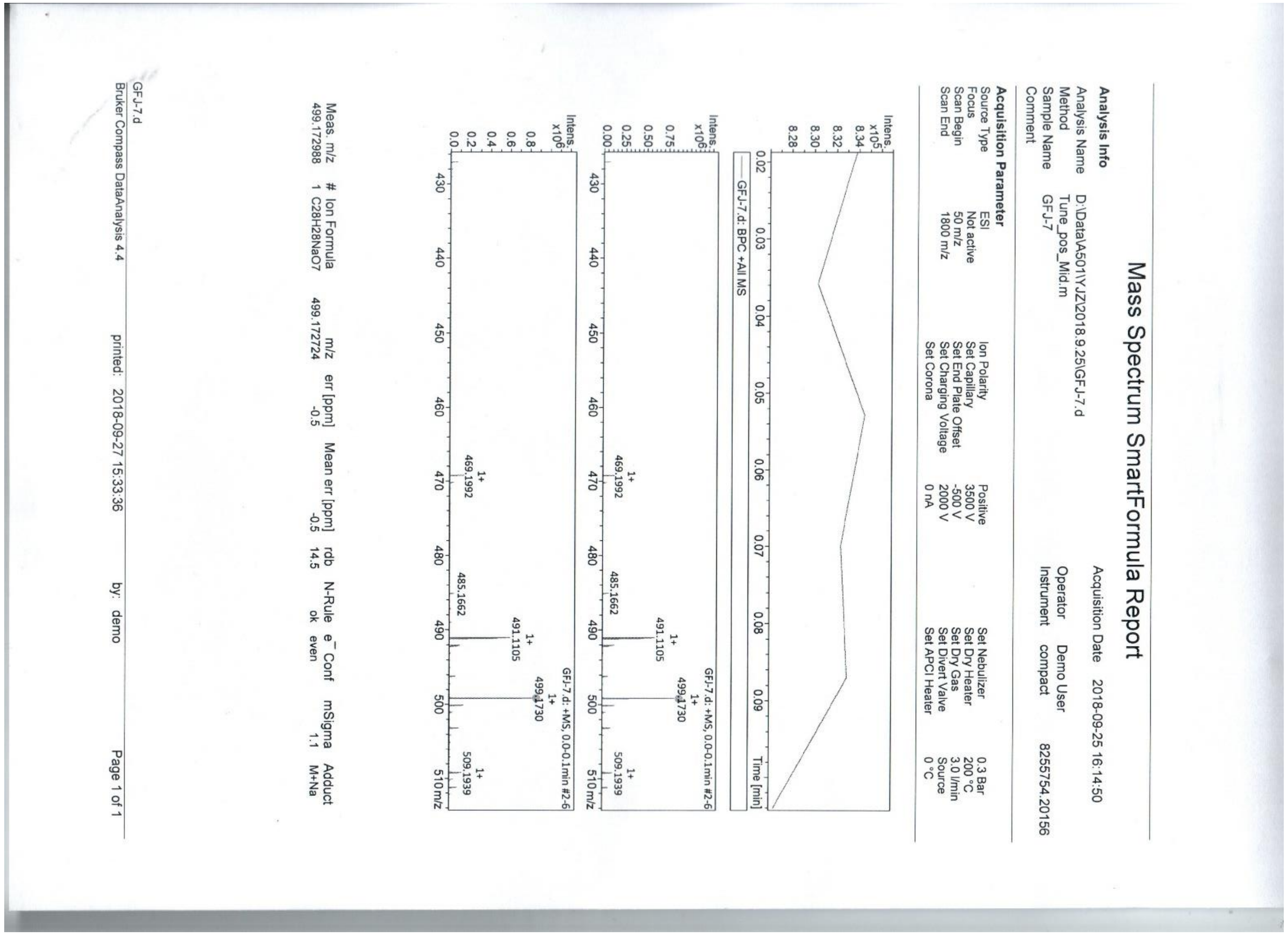

Figure S1. HRESIMS spectrum of **1**

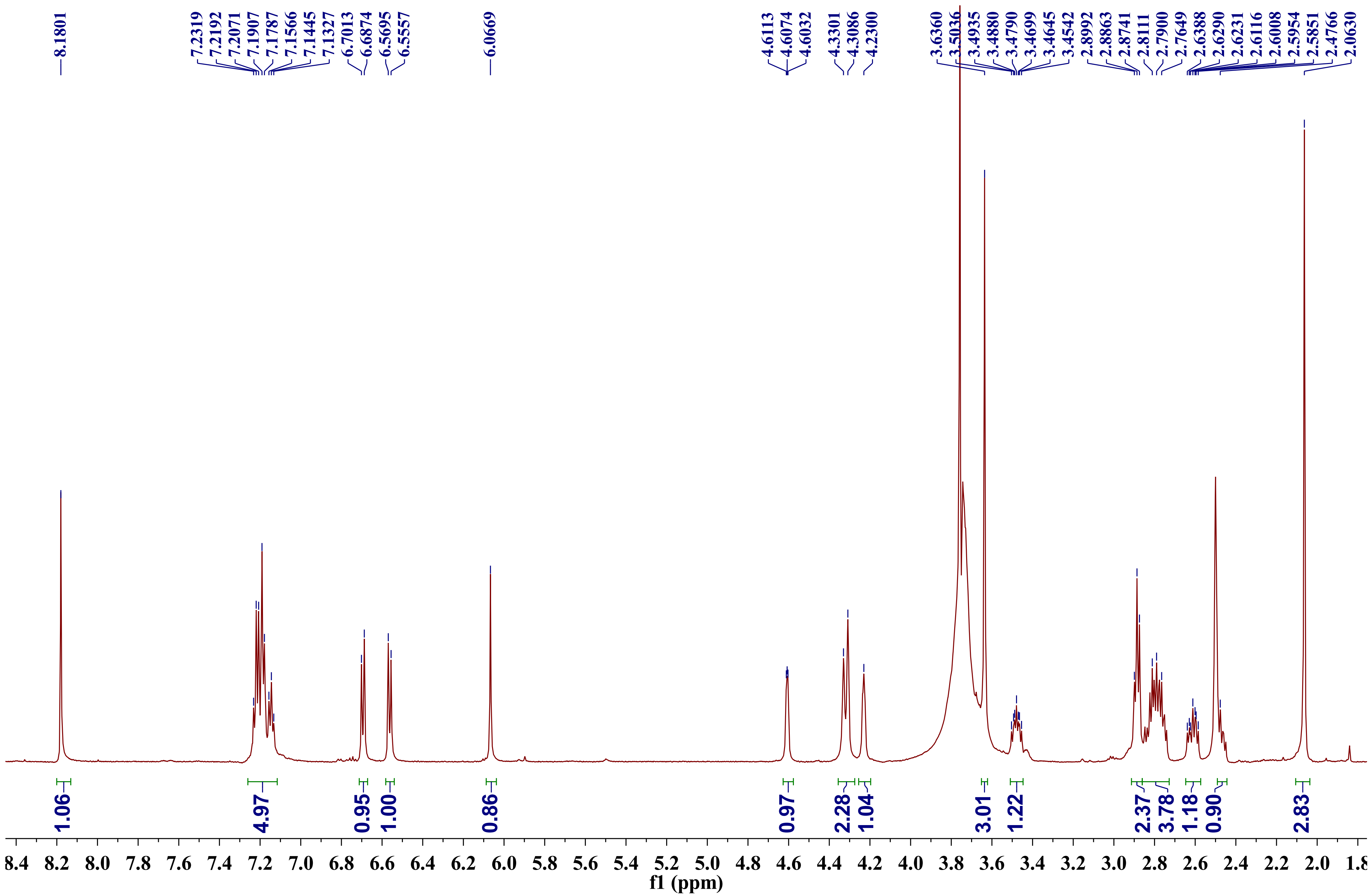

Figure S2. ^{1}H NMR (600 MHz, DMSO-d_6) spectrum of **1**

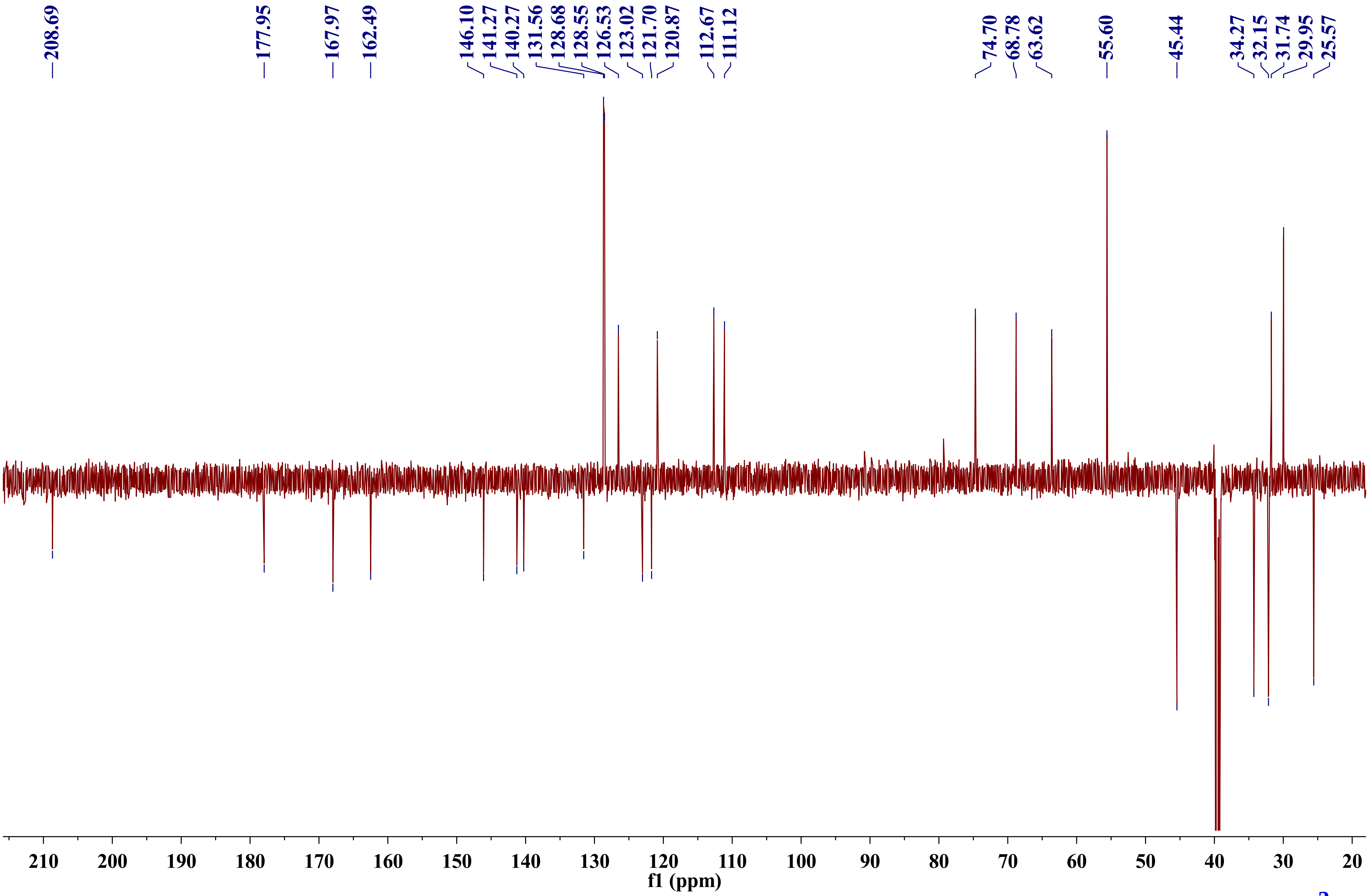

Figure S3. DEPTQ (150 MHz, DMSO-d_6) spectrum of **1**

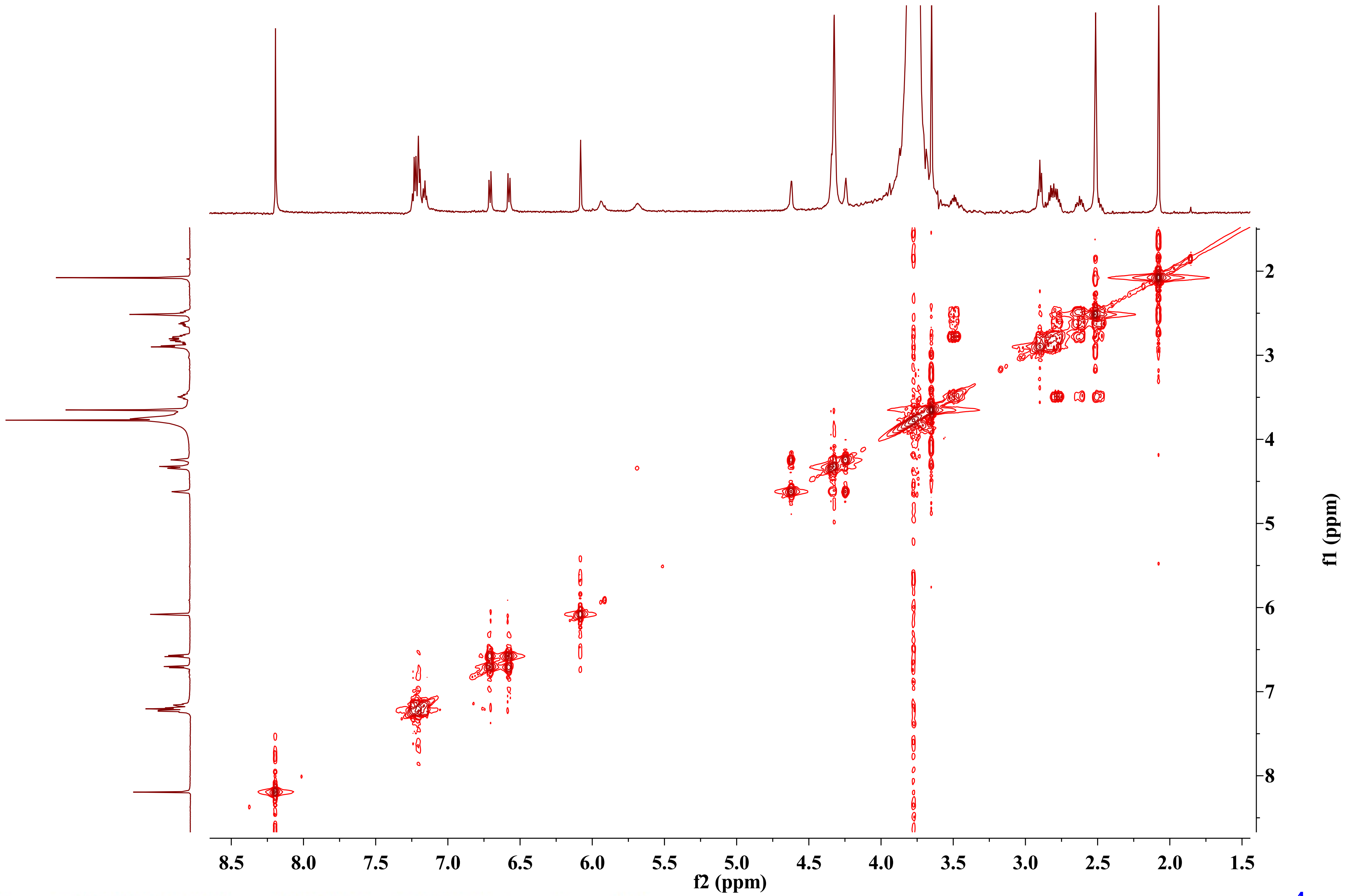

Figure S4. HSQC (600 and 150 MHz, DMSO-d_6) spectrum of **1**

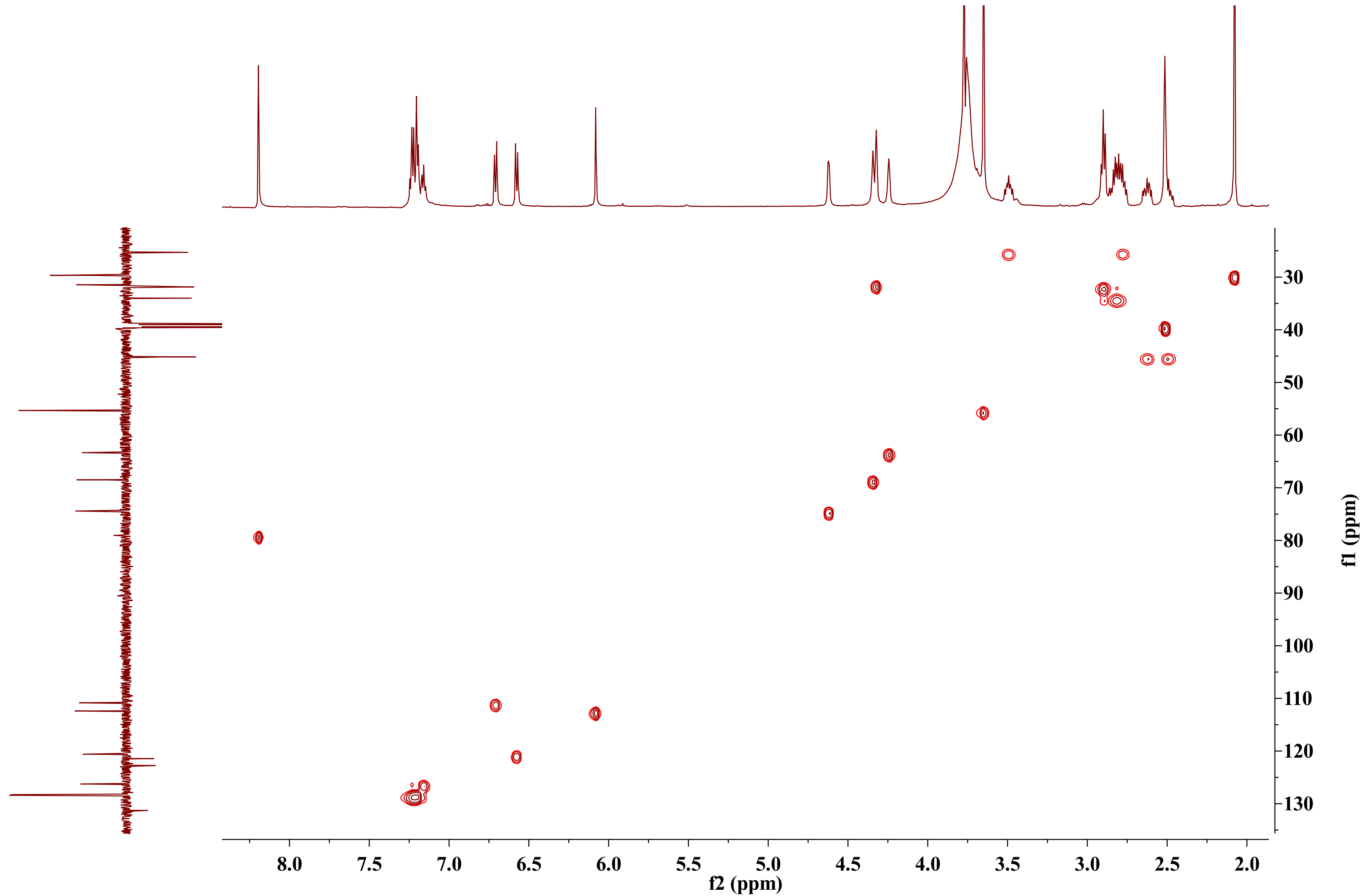

Figure S5. HSQC (600 and 150 MHz, DMSO-d_6) spectrum of **1**

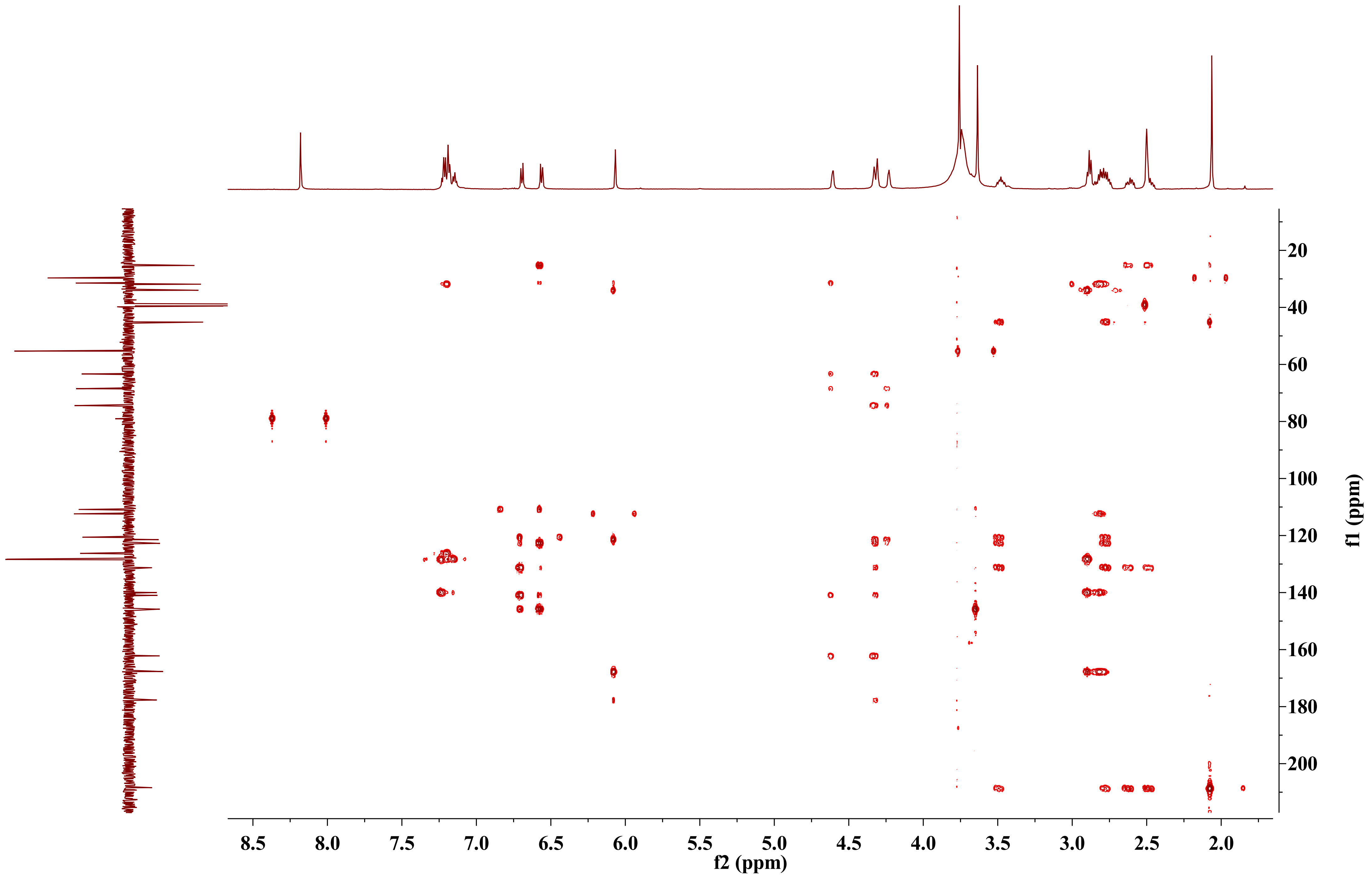

Figure S6. HMBC (600 and 150 MHz, DMSO-d_6) spectrum of **1**

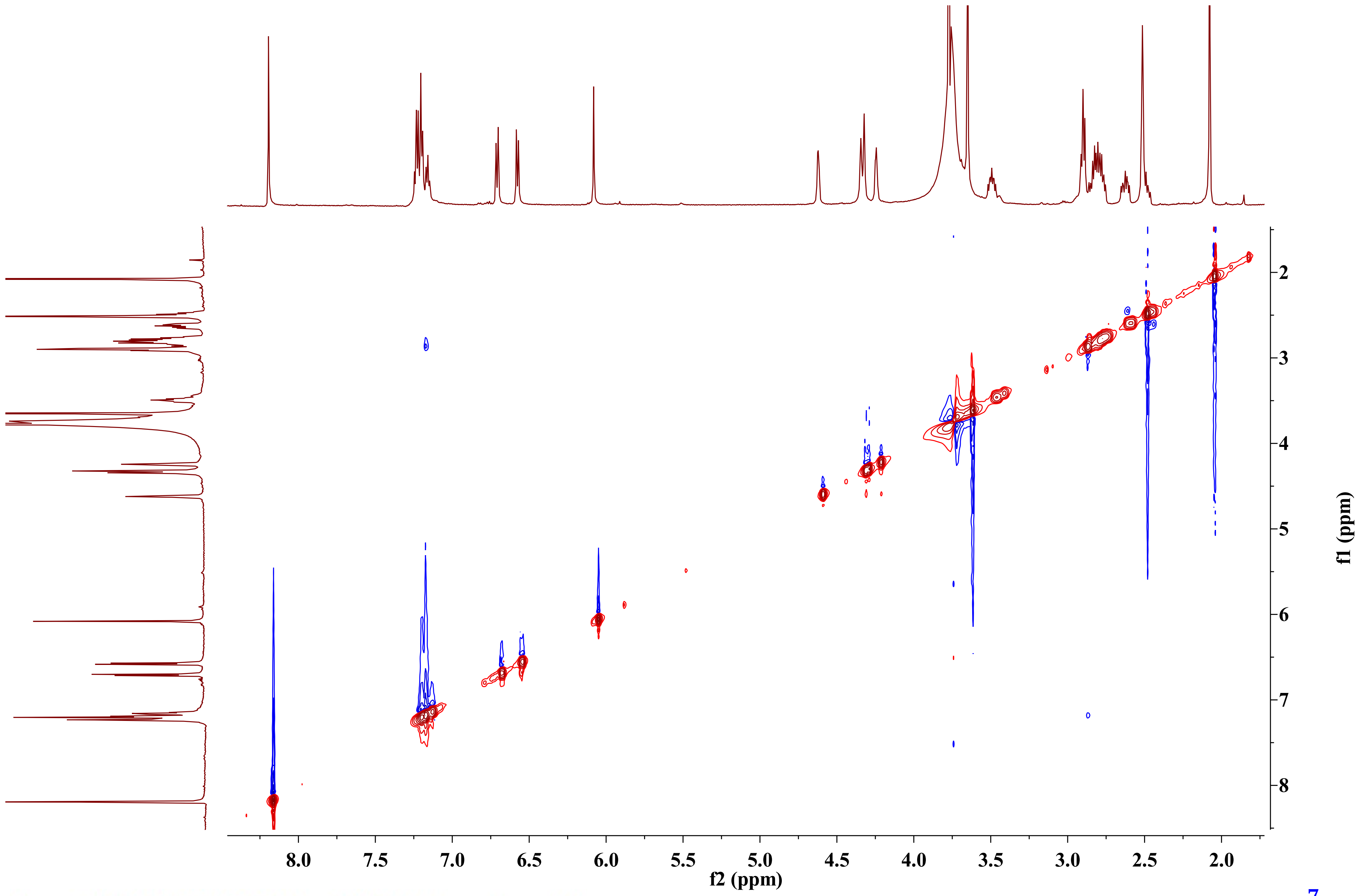

Figure S7. ROESY (600 MHz, DMSO-d_6) spectrum of **1**

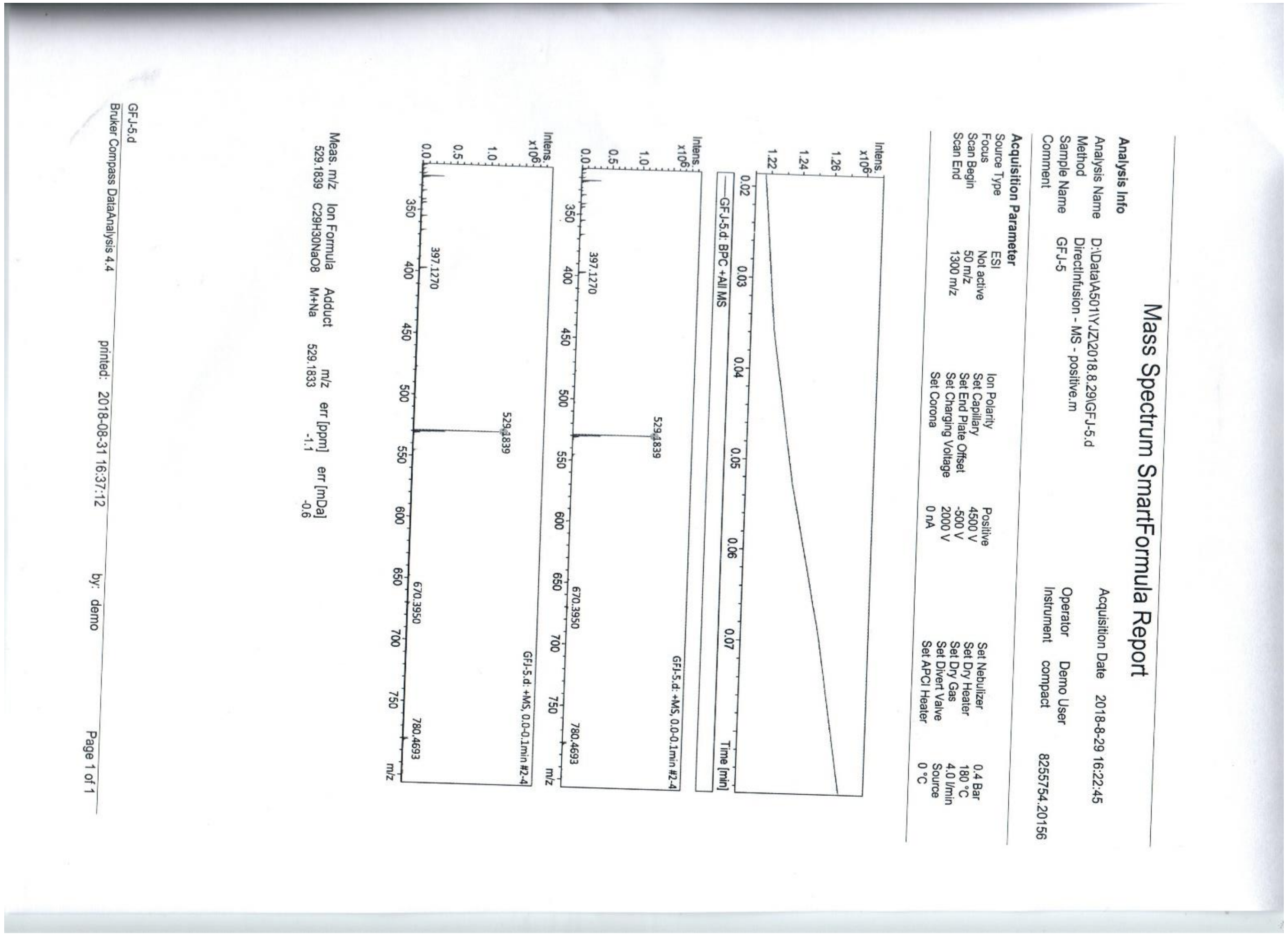

Figure S8. HRESIMS spectrum of **2**

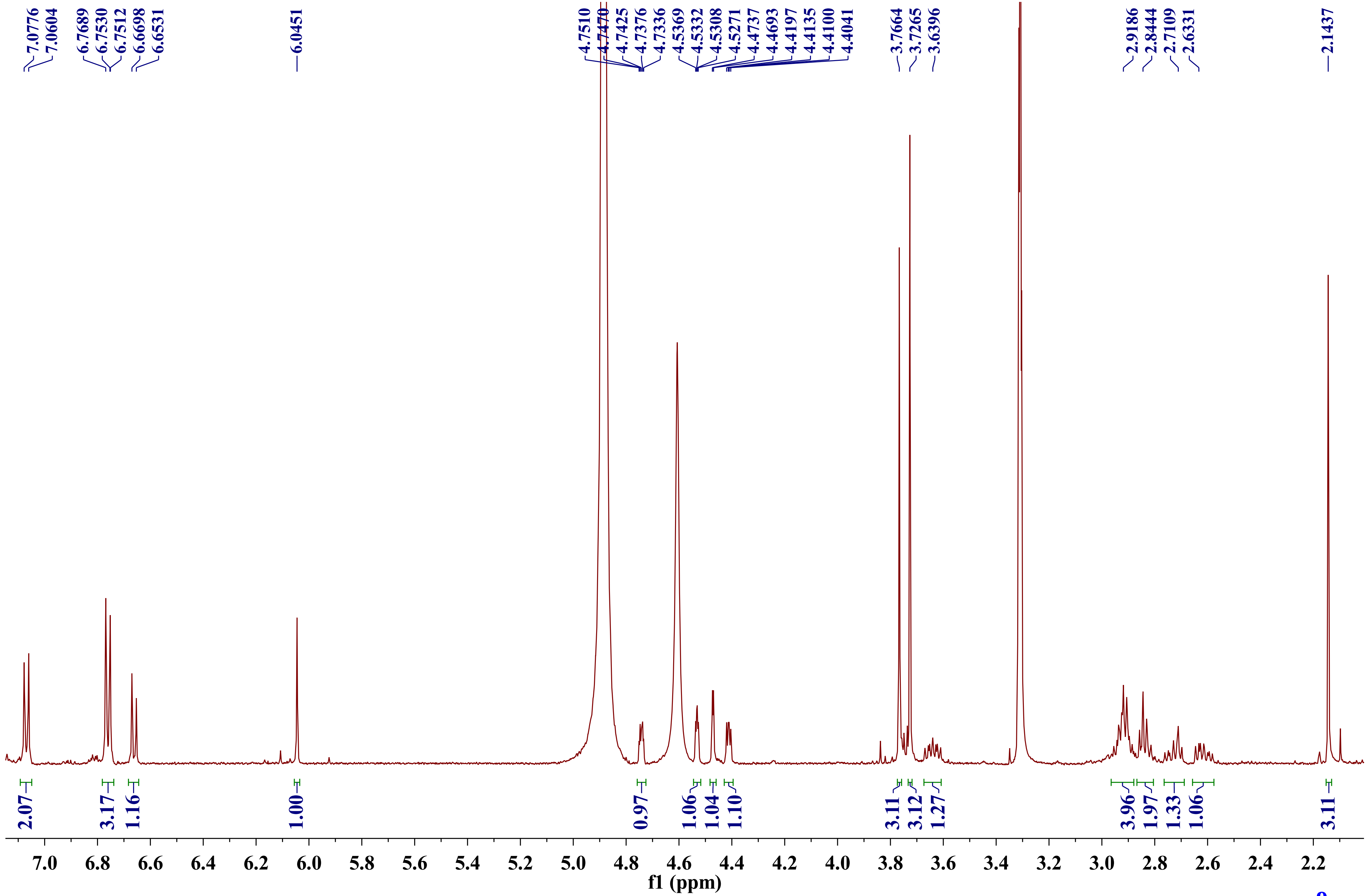

Figure S9. ^{1}H NMR (600 MHz, methanol-d_4) spectrum of **2**

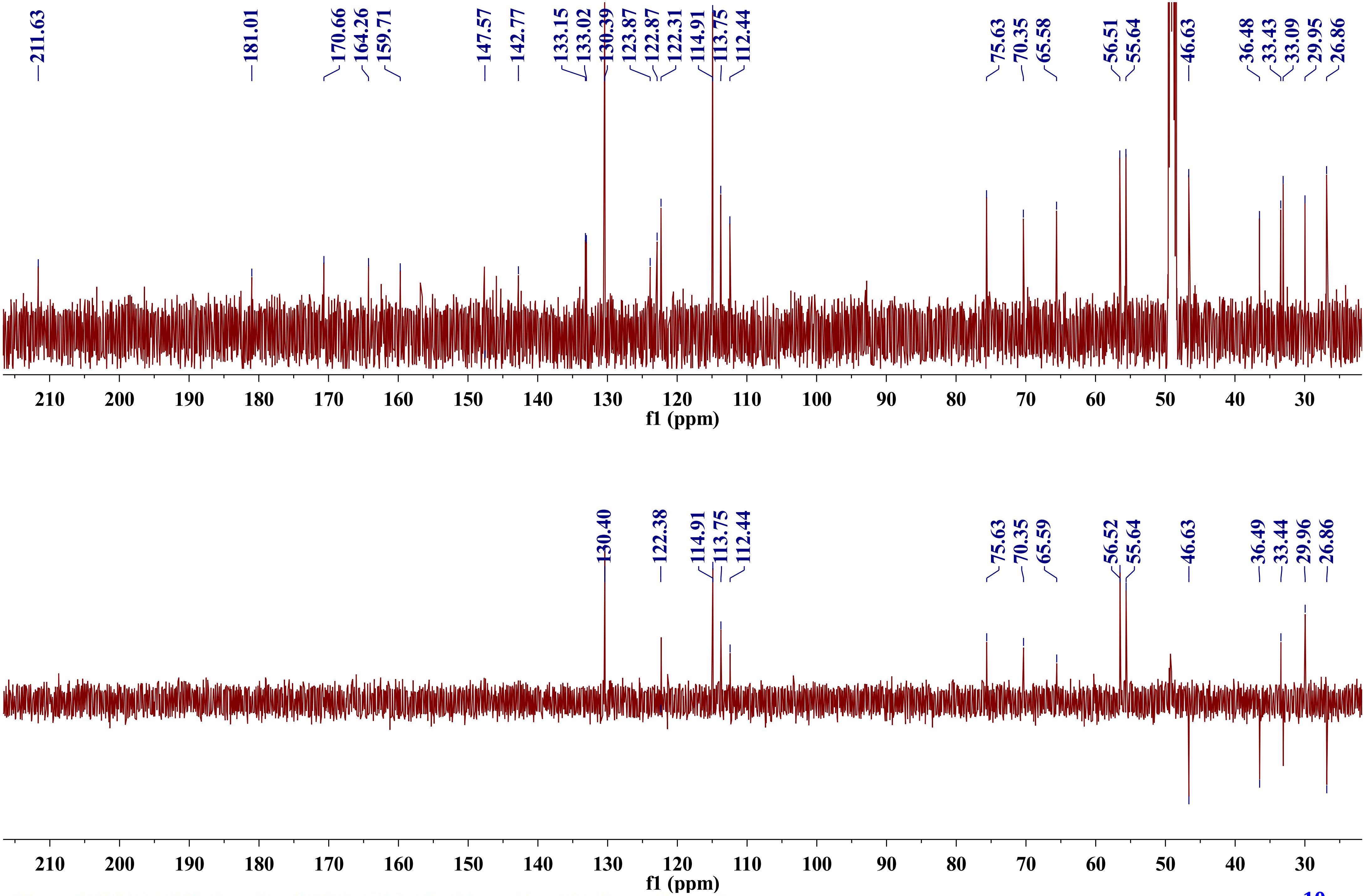

Figure S10. ^{13}C NMR and DEPT-135 (150 MHz, methanol-d_4) spectrum of **2**

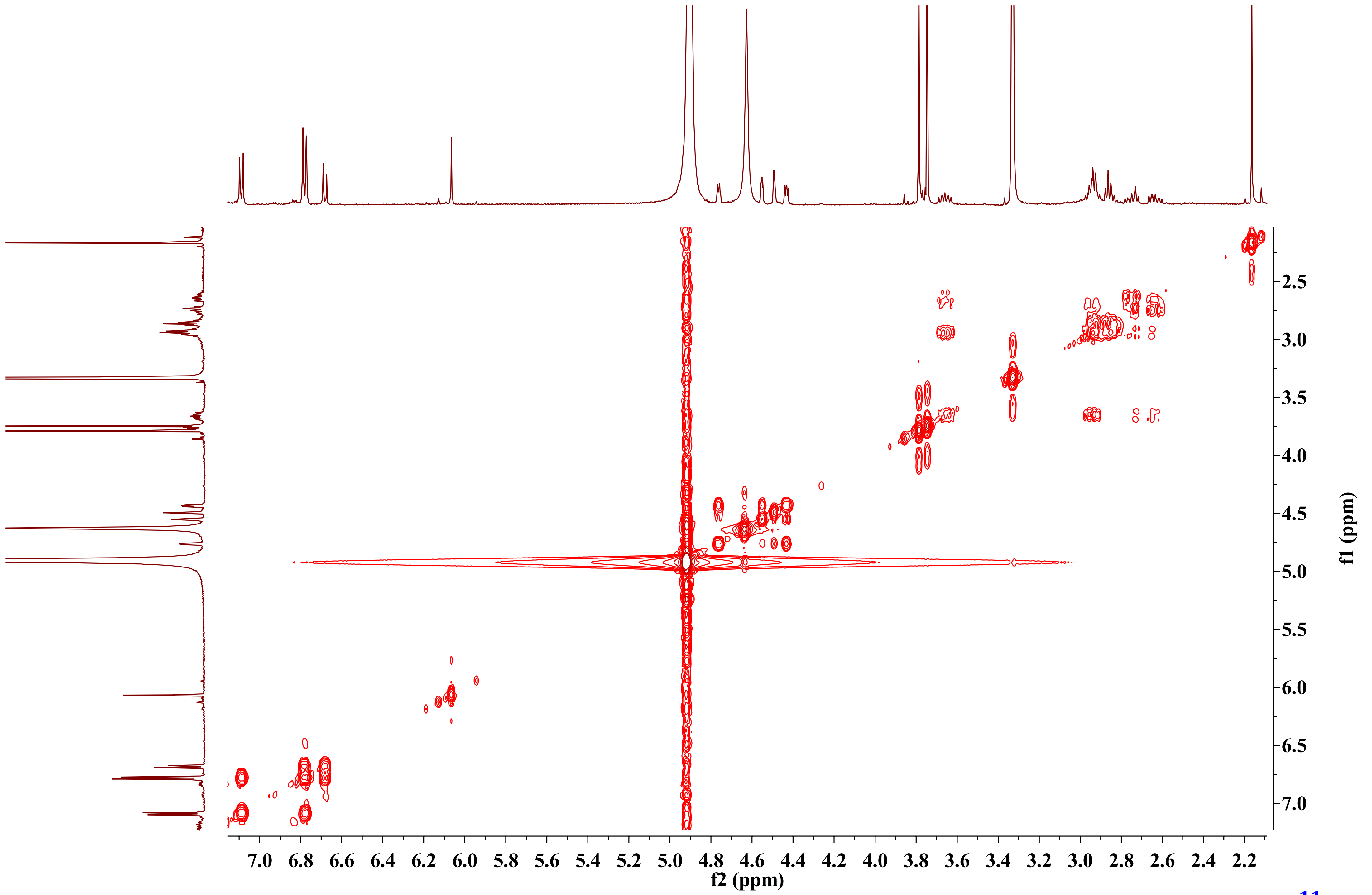

Figure S11. ^{1}H-^{1}H COSY (600 MHz, methanol-d_4) spectrum of **2**

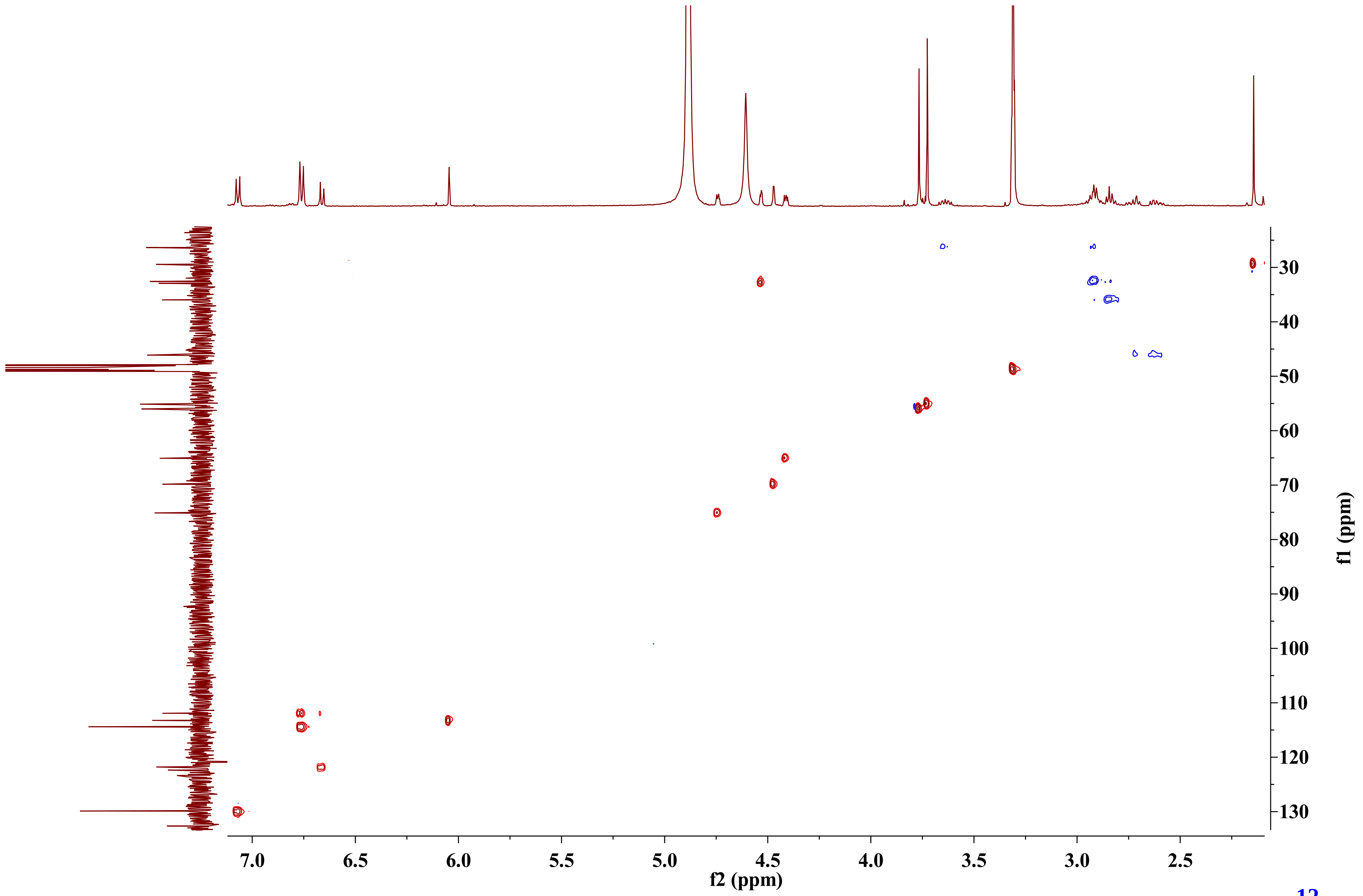

Figure S12. HSQC (600 and 150 MHz, methanol-d_4) spectrum of **2**

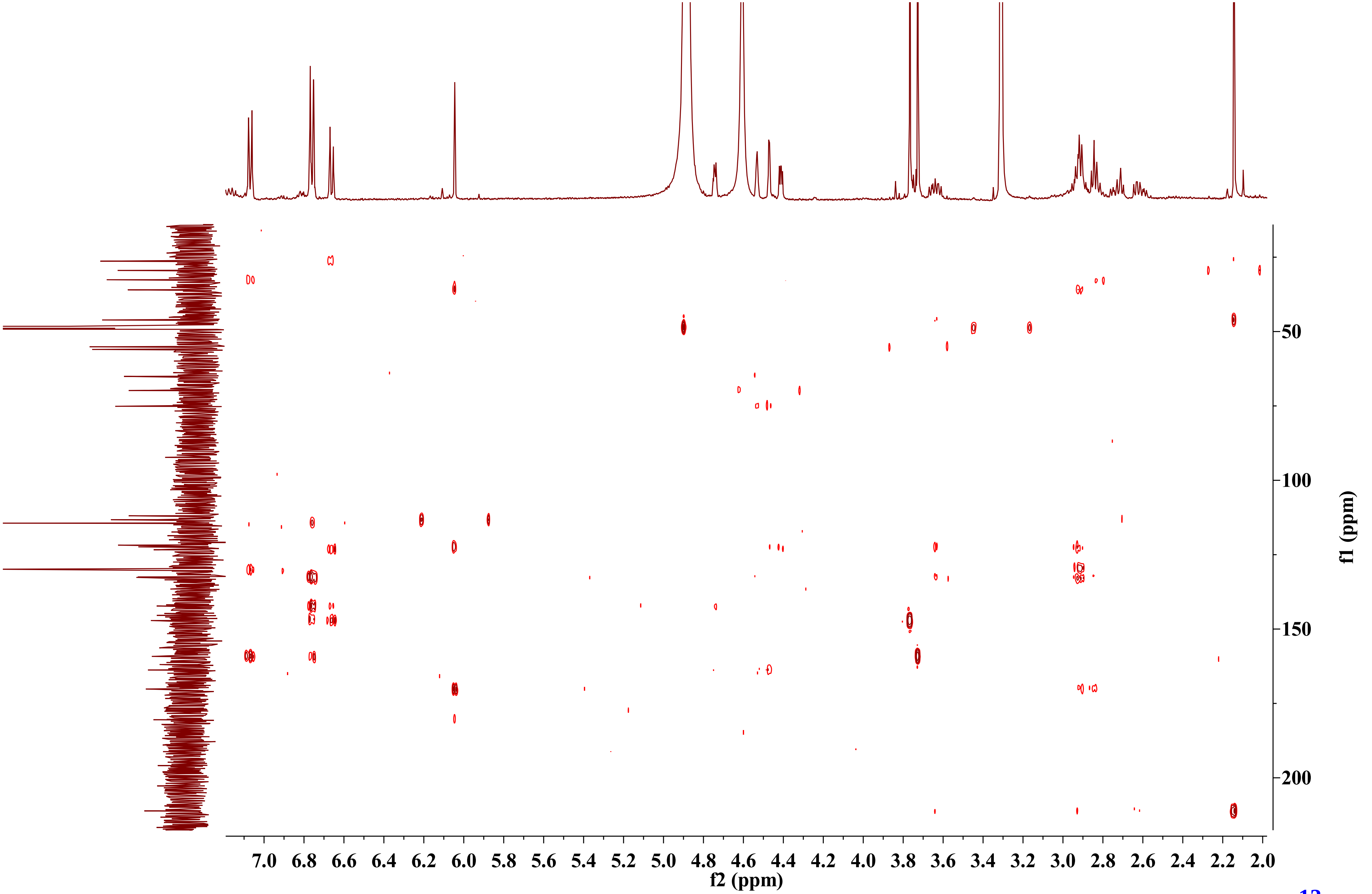

Figure S13. HMBC (600 and 150 MHz, methanol-d_4) spectrum of **2**

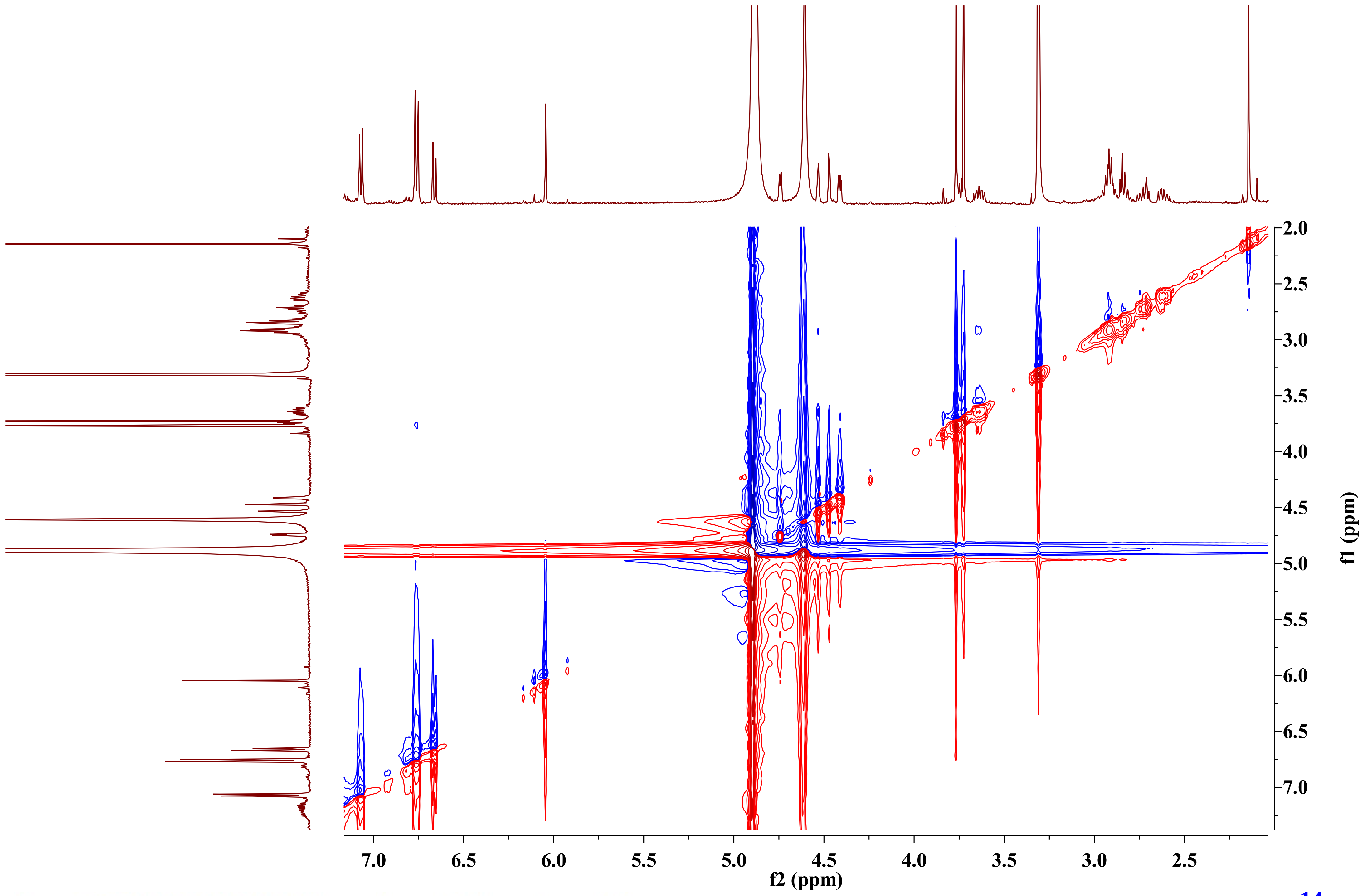

Figure S14. ROESY (600 MHz, methanol-*d*$_4$) spectrum of **2**

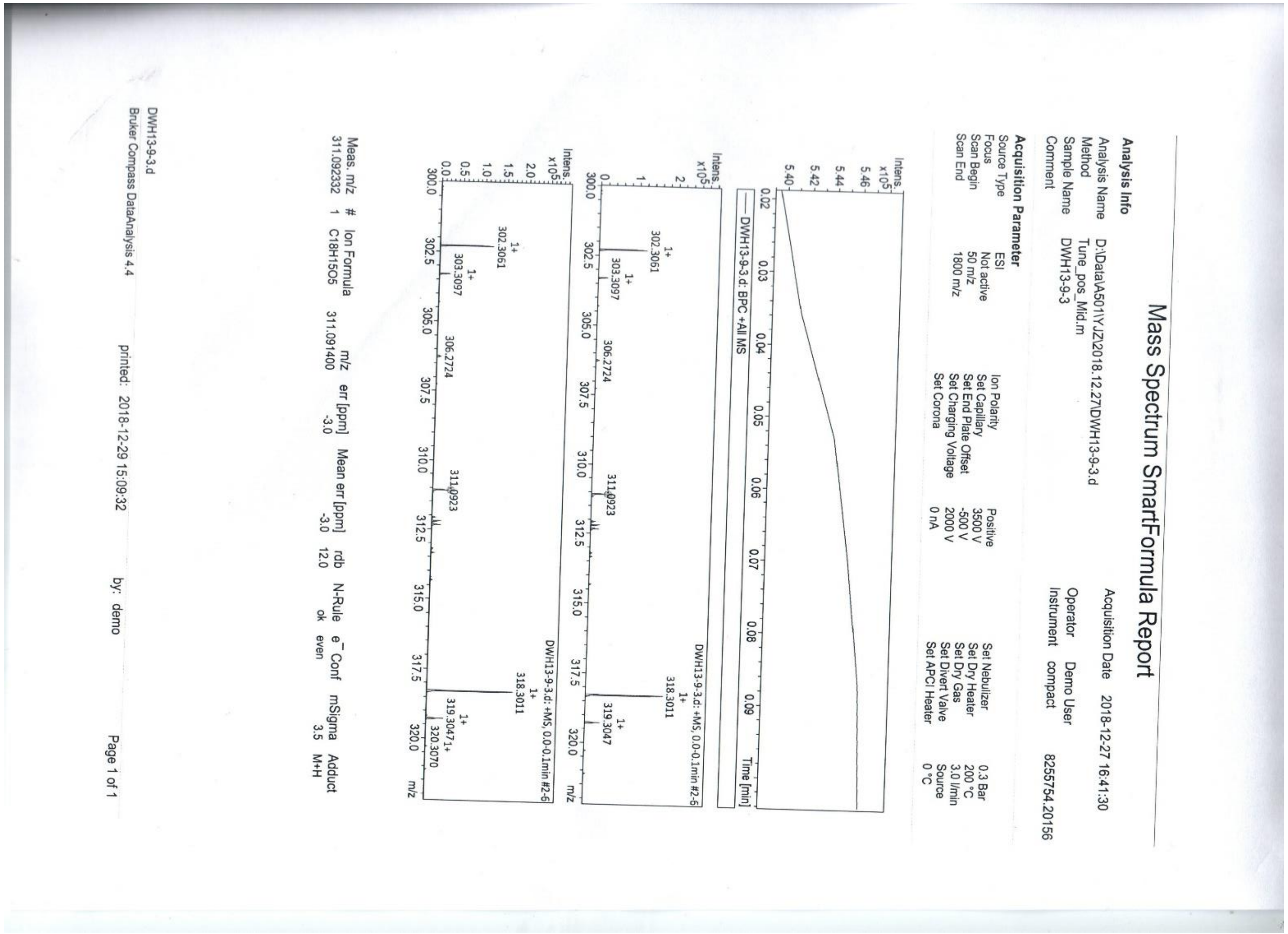

Figure S15. HRESIMS spectrum of **3**

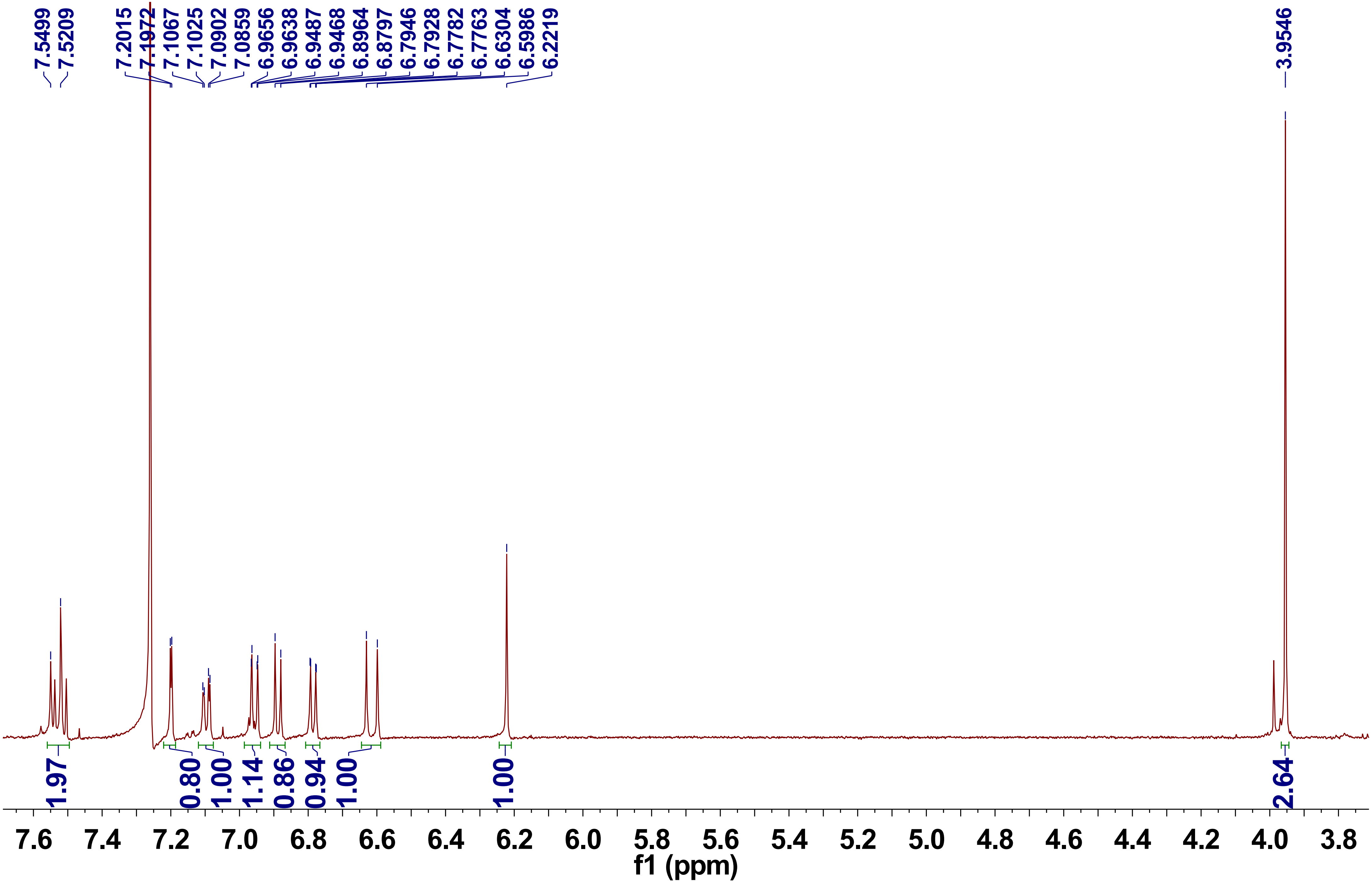

Figure S16. ^{1}H NMR (500 MHz, $CDCl_3$) spectrum of **3**

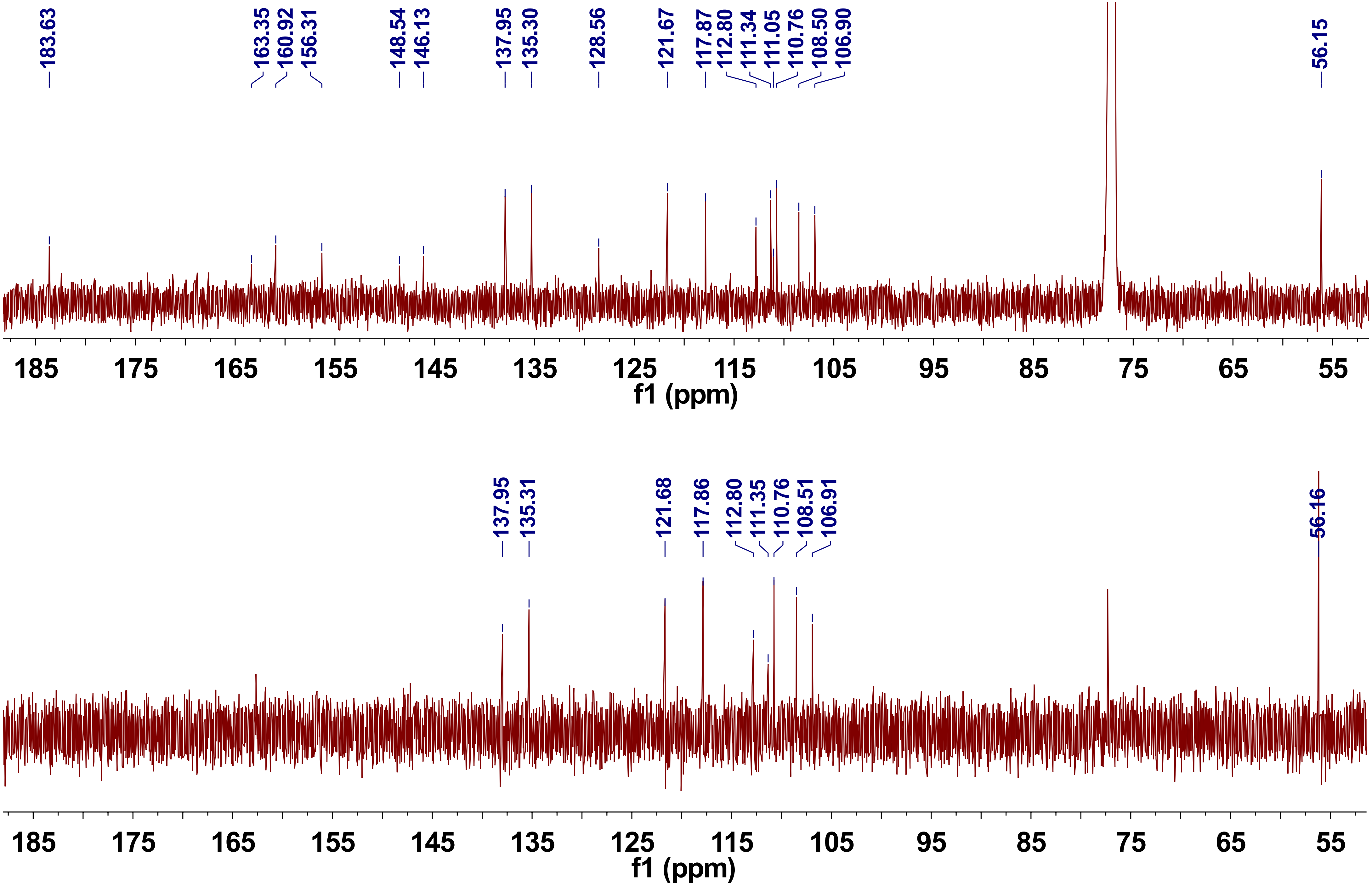

Figure S17. ^{13}C NMR and DEPT-135 (125 MHz, $CDCl_3$) spectrum of **3**

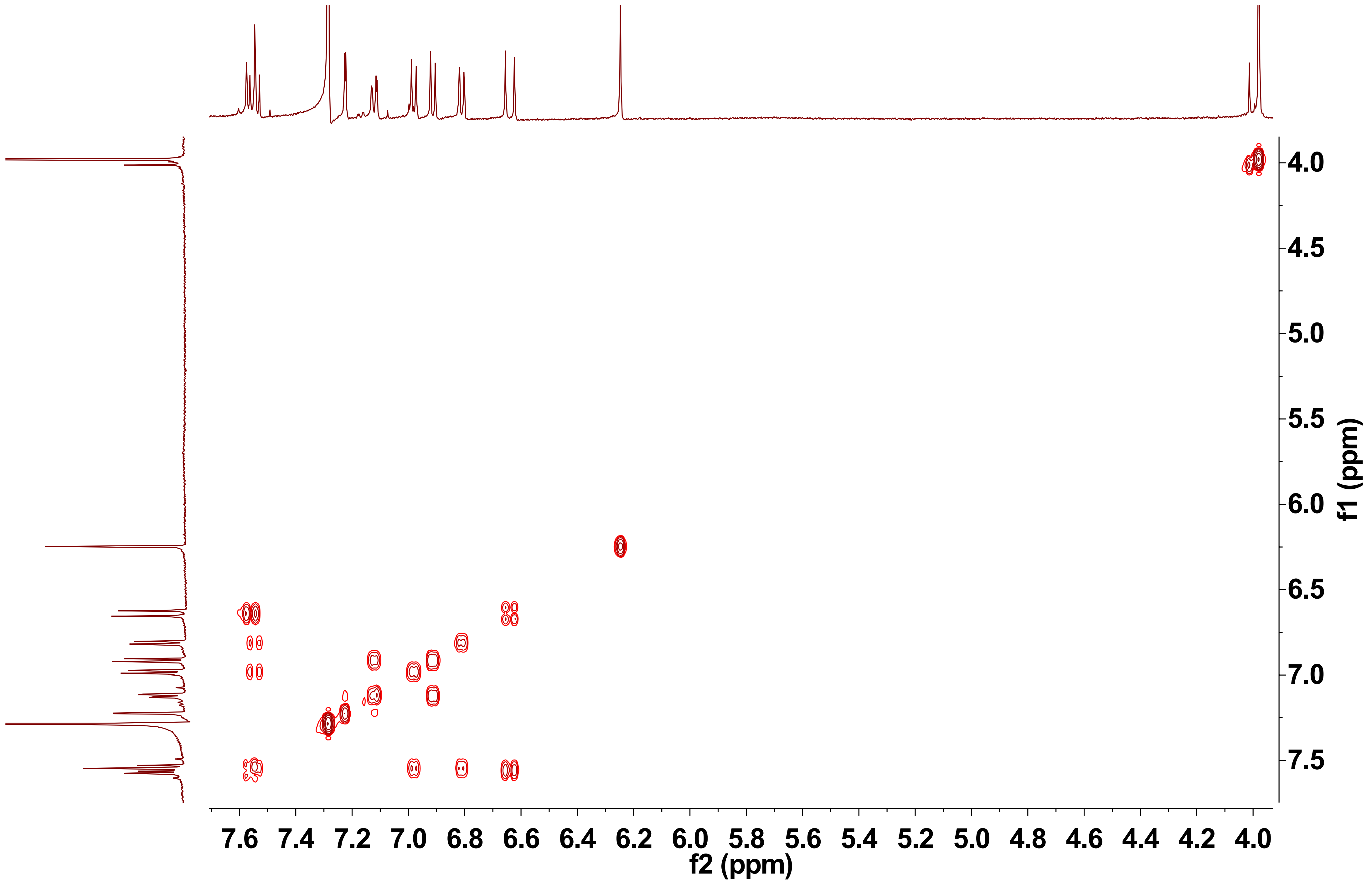

Figure S18. ^1H-^1H COSY (500 MHz, $CDCl_3$) spectrum of **3**

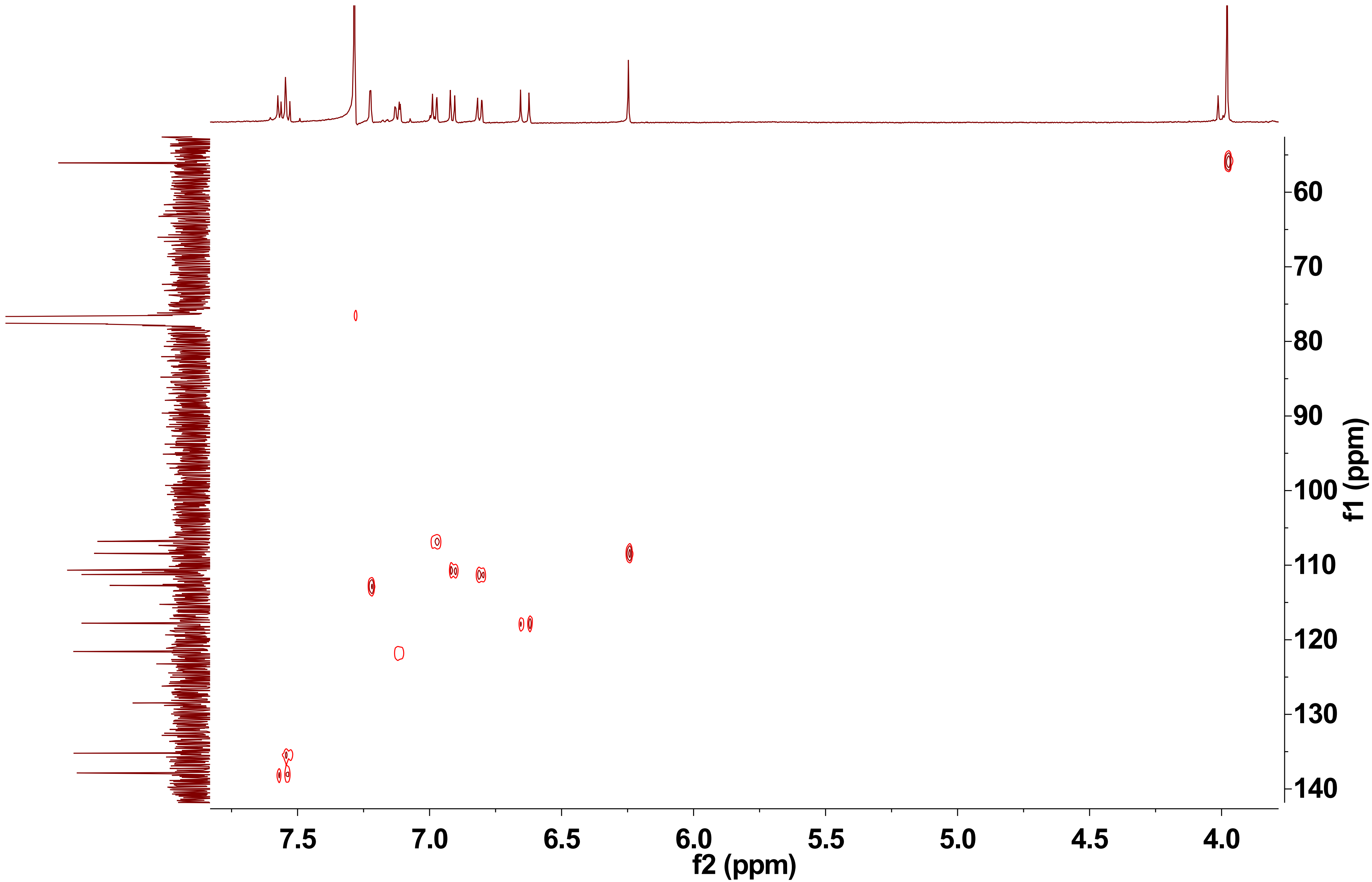

Figure S19. HSQC (500 and 125 MHz, $CDCl_3$) spectrum of **3**

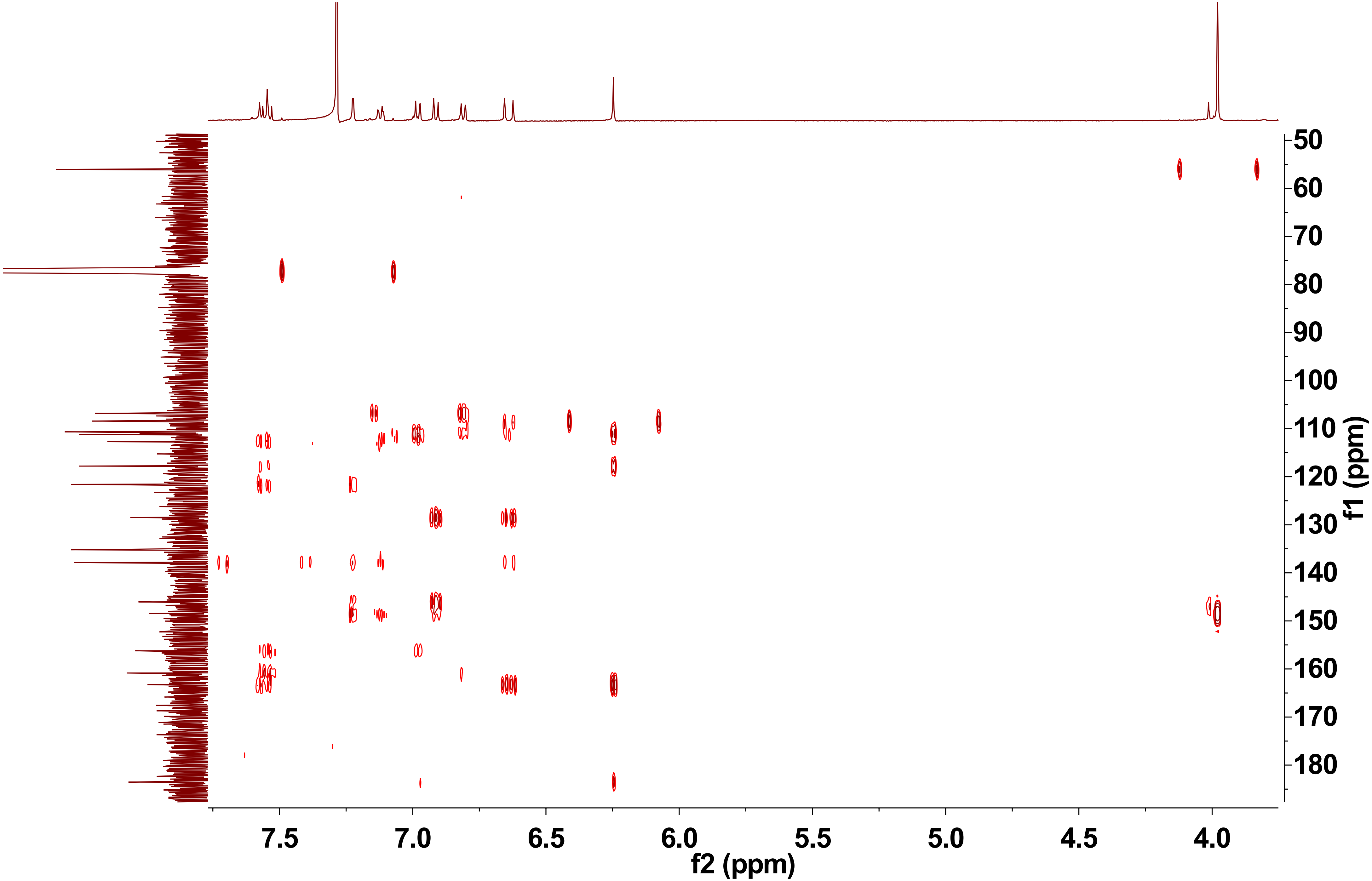

Figure S20. HMBC (500 and 125 MHz, $CDCl_3$) spectrum of **3**

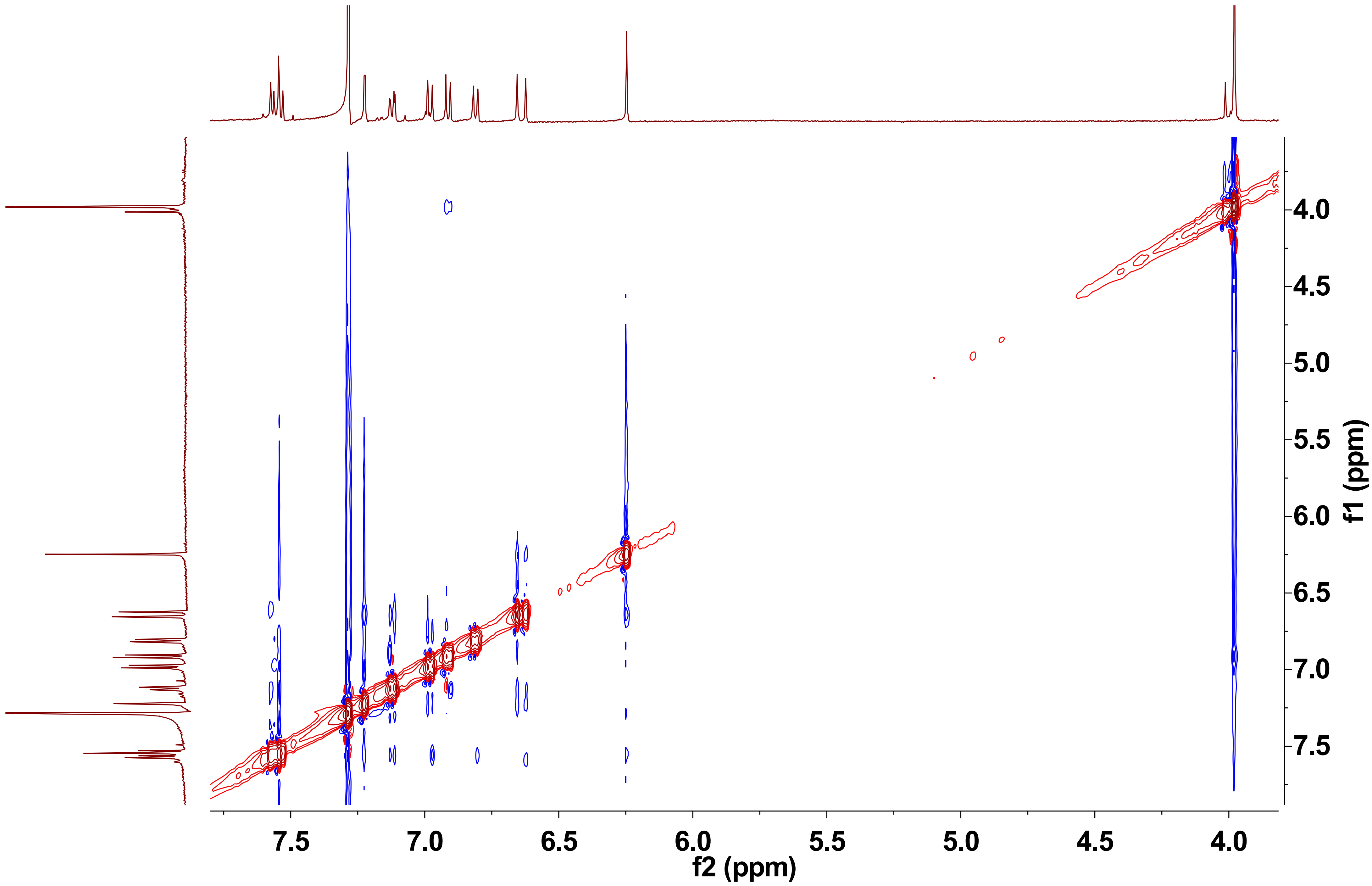

Figure S21. ROESY (500 MHz, $CDCl_3$) spectrum of 3
